# Expression and function of voltage gated proton channels (H_v_1) in MDA-MB-231 cells

**DOI:** 10.1371/journal.pone.0227522

**Published:** 2020-05-06

**Authors:** Dan J. Bare, Vladimir V. Cherny, Thomas E. DeCoursey, Abde M. Abukhdeir, Deri Morgan

**Affiliations:** 1 Department of Physiology & Biophysics, Rush University, Chicago, IL, United States of America; 2 Department of Internal Medicine, Rush University Medical Center, Chicago, IL, United States of America; 3 Department of Radiation Oncology, University of Kansas Medical Center, Kansas City, MO, United States of America; Duke University School of Medicine, UNITED STATES

## Abstract

Expression of the voltage gated proton channel (H_v_1) as identified by immunocytochemistry has been reported previously in breast cancer tissue. Increased expression of H_V_1 was correlated with poor prognosis and decreased overall and disease-free survival but the mechanism of its involvement in the disease is unknown. Here we present electrophysiological recordings of H_V_1 channel activity, confirming its presence and function in the plasma membrane of a breast cancer cell line, MDA-MB-231. With western blotting we identify significant levels of H_V_1 expression in 3 out of 8 “triple negative” breast cancer cell lines (estrogen, progesterone, and HER2 receptor expression negative). We examine the function of H_V_1 in breast cancer using MDA-MB-231 cells as a model by suppressing the expression of H_V_1 using shRNA (knock-down; KD) and by eliminating H_V_1 using CRISPR/Cas9 gene editing (knock-out; KO). Surprisingly, these two approaches produced incongruous effects. Knock-down of H_V_1 using shRNA resulted in slower cell migration in a scratch assay and a significant reduction in H_2_O_2_ release. In contrast, H_V_1 Knock-out cells did not show reduced migration or H_2_O_2_ release. H_V_1 KO but not KD cells showed an increased glycolytic rate accompanied by an increase in p-AKT (phospho-AKT, Ser473) activity. The expression of CD171/LCAM-1, an adhesion molecule and prognostic indicator for breast cancer, was reduced in H_V_1 KO cells. When we compared MDA-MB-231 xenograft growth rates in immunocompromised mice, tumors from H_V_1 KO cells grew less than WT in mass, with lower staining for the Ki-67 marker for cell proliferation rate. Therefore, deletion of H_V_1 expression in MDA-MB-231 cells limits tumor growth rate. The limited growth thus appears to be independent of oxidant production by NADPH oxidase molecules and to be mediated by cell adhesion molecules. Although H_V_1 KO and KD affect certain cellular mechanisms differently, both implicate H_V_1-mediated pathways for control of tumor growth in the MDA-MB-231 cell line.

## Introduction

The voltage gated proton channel (H_V_1), part of the superfamily of voltage-gated membrane proteins, is a membrane bound 273 amino acid protein that forms a pH- and voltage-gated ion channel that conducts protons [1, 2]. It forms a dimer in the membrane in which each monomer has four membrane spanning helices (S1-S4) and each monomer has its own proton-conducting pathway [3–5]. When the channel opens it is perfectly selective for protons [6–8]. The channel senses the pH gradient across the cell membrane and opens when the electrochemical gradient for H^+^ is outward, resulting in acid extrusion that raises pH of the cytosol [9]. In cell membranes H_V_1 extrudes H^+^ electrogenically, causing membrane hyperpolarization. During the respiratory burst of phagocytes, it facilitates and sustains the activity of the enzyme NADPH oxidase by compensating for both pH and membrane potential changes that would otherwise inhibit the enzyme’s function [10–13]. A close functional relationship with NADPH oxidase is also seen in B cell receptor signaling [14] and in pathophysiological states in ischemic stroke where NADPH oxidase in microglia contributes to bystander injury facilitated by H_V_1 [15]. Important physiological effects of H_v_1 on cytosolic pH have also been demonstrated during histamine release by human basophils [16] and in sperm where it contributes to capacitation and motility [17, 18]. In mammals, only a single gene codes for H_V_1 although the channel protein is truncated in sperm and in chronic lymphocytic leukemia through post-translational processing [19, 20].

H_V_1 protein expression was reported in certain types of breast cancer cells and inhibition of the channel expression by siRNA or shRNA was found to reduce migration, lower the release of matrix metalloproteinase enzymes and produce smaller tumors in a mouse model [21]. A follow-up clinical study showed a correlation between the expression levels of H_V_1 and worse prognosis, lower recurrence-free survival, and poor disease scores [22]. However, the mechanisms by which H_V_1 regulate breast cancer cell growth are not known.

One hallmark of cancers is the disordered metabolism seen in a large number of tumors that upregulate the uptake and use of glucose as a source of energy [23, 24]. Many cancer cells utilize glycolysis as the predominant energy pathway, causing local lactic acidosis either due to the tumor microenvironment being hypoxic and requiring anaerobic fuel sources or because the cells have become reprogrammed to utilize glycolysis even in the presence of adequate oxygen (the Warburg effect) [25, 26]. The increased glycolysis and resulting acidosis of the surrounding tissue causes environmental pressure on both cancer cells and the surrounding tissue. Cancer cells adapt to this change by upregulating a number of proton transporters including H^+^-ATPases, Na^+^/H^+^ antiport, carbonic anhydrase, bicarbonate transporters and monocarboxylate transporters resulting in a cytosolic pH (pH_i_) that is higher (pH_i_ = 7.2–7.4) than the surrounding milieu (pH_o_ ~6.9) [27–29].

The specific properties of H_V_1 in breast cancer cells run contrary to the action of pumps and transporters that are upregulated in cancer because it moves protons only down the electrochemical gradient, whereas energy-consuming transport is required to invert the pH gradient. This feature must be considered in order to establish how H_V_1 modulates cancer cell growth. Here we present evidence that the proton channel H_V_1 contributes to *in vivo* tumor growth through mechanisms that are independent of reactive oxygen species (ROS) production.

## Methods

### Cell lines and cell culture

Cells were obtained either from ATCC (American Type Culture Collection; HEK297, MDA-MB-231, MCF-7, MDA-MB-468, MDA-MB-436, SKBR3, BT-474, MCF-10A, BT-20, BT-549, Hs578t and MCF-10A) or from Asterand Bioscience (Sum-159PT, Sum-229PE). Cells were grown in a Forma Scientific water-jacketed incubator at 37°C with 5% CO_2_. DMEM media supplemented with 10% FBS, GlutaMAX, and 25 mM HEPES (Invitrogen, USA) was used for MDA-MB-231, MCF-7, Hs578t, BT-20, MDA-MB-436, MDA-MB-468, and Hs578t cell lines. Ham’s F-12 medium with 10% fetal bovine serum, 10 mM HEPES, 1 μg/ml hydrocortisone and 5 μg/ml bovine insulin was used to grow SUM-229PE and Sum-159PT. DMEM/F12 with 5% fetal horse serum, 2 ng/ml EGF, 0.5 mg/ml hydrocortisone, 100 ng/ml cholera toxin, and 10 μg/ml insulin was used for MCF-10A cells. All media were supplemented with penicillin/streptomycin (Invitrogen) at 1000/100 units/ml.

Cells lines were utilized for fewer than 20 passes. All extracts and transfections from cells were taken from sub-confluent cells in their logarithmic phase of growth unless stated.

### Electrophysiology

Cells were grown to ~80% confluence in 35 mm cultures dishes at 37°C in 5% CO_2_. To detach the cells, media was removed and warm trypsin (0.25% in EGTA; GIBCO) was added for 1–2 min. Adding medium stopped the trypsinization, then cells were centrifuged for 4 min at 200 x g and resuspended in fresh medium. The cells were plated onto glass cover slips at low density for patch clamp recording.

Micropipettes were pulled using a Flaming Brown automatic pipette puller (Sutter Instruments, San Rafael, CA) from Custom 8520 Patch Glass (equivalent to Corning 7052 glass; Harvard Apparatus, Holliston, MA), coated with Sylgard 184 (Dow Corning Corp., Midland, MI), and heat polished to a tip resistance ranging typically 3-10 MΩ measured with highly buffered tetramethylammonium^+^ (TMA^+^) containing pipette solutions. Electrical contact with the pipette solution was achieved by a thin sintered Ag-AgCl pellet (In Vivo Metric Systems, Healdsburg, CA) attached to a Teflon-encased silver wire, or simply a chlorided silver wire. A reference electrode made from a Ag-AgCl pellet was connected to the bath through an agar bridge made with Ringer's solution (in mM: 160 NaCl, 4.5 KCl, 2 CaCl_2_, 1 MgCl_2_, 5 HEPES, pH 7.4). The current signal from the patch clamp (EPC-9 from HEKA Instruments Inc., Holliston, MA, or Axopatch 200B from Axon Instruments, Foster City, CA) was recorded and analyzed using Pulse and PulseFit software (HEKA), or pCLAMP software (molecular Devices, San Jose, CA) supplemented by Sigmaplot (SPSS Inc., Chicago, IL). Seals were formed with Ringer's solution in the bath, and the potential zeroed after the pipette was in contact with the cell. Current records are displayed without correction for liquid junction potentials.

The whole-cell configuration of the patch-clamp technique was used. Bath and pipette solutions were used interchangeably. These solutions contained (in mM) 2 MgCl_2_, 1 EGTA, 80–100 buffer, 75–120 TMA^+^ CH_3_SO_3_^–^ (adjusted to bring the osmolality to ~300 mOsm), and were titrated using TMAOH. Buffers with p*K*_a_ near the desired pH were used: Homo-PIPES for pH 4.5–5.0, MES for pH 5.5–6.0, BisTris for pH 6.5, BES for pH 7.0, HEPES for pH 7.5, tricine for pH 8.0, and CHES for pH 9.0. Experiments were performed at room temperature (~20–25°C). Current records are shown without leak correction.

Reversal potentials (*V*_rev_) in most cases were determined from the direction and amplitude of tail current relaxation over a range of voltages, following a prepulse that activated the proton conductance, *g*_H_. Currents were fitted with a single exponential to obtain the activation time constant (*τ*_act_) and the fitted curve was extrapolated to infinite time to obtain the “steady-state” current amplitude (*I*_H_), from which the *g*_H_ was calculated as *g*_H_ = *I*_H_/(*V*-*V*_rev_). Thus, we assume that the time dependent component is due to H^+^ current and that time independent current represents leak. Because of the strong voltage dependence of activation kinetics, we frequently applied longer pulses near threshold voltages, and shorter pulses for large depolarizations in order to resolve kinetics and avoid proton depletion associated with large H^+^ flux.

### Membrane protein isolation and western blotting

Cells growing exponentially in culture were scraped using a cell scraper and pelleted by centrifugation at 200 x g for 4 min at 4°C. Cells were resuspended in PBS and washed twice by centrifugation and resuspension. Cell pellets were resuspended in ice cold homogenization buffer (140 mM Tris-HCl, EGTA 10 mM) supplemented with protease inhibitors (HALT cocktail -100X; Thermo Fisher Scientific, Waltham, MA). The cells were lysed by being drawn through a 27-gauge needle 15–20 times using a 1 ml syringe while being kept at 4°C. The resulting cell lysate was centrifuged at 16,000 x g for 30 min. The supernatant was discarded and the resulting membrane pellet was resuspended in fresh buffer with protease inhibitors. The protein concentration of the membrane samples was determined using a micro BSA protein assay kit (Thermo Fisher Scientific) and the samples were frozen at -80°C until further use. Cell membrane samples were rapidly thawed, protein concentrations were normalized by adding buffer (between 20–40 μg/well, with concentrations constant on the same blot), and 250 μl of Laemmli sample buffer (Bio-Rad Labs, Hercules, CA) was added. This solution was boiled for 10 min at 100°C and protein isolates were resolved using SDS-PAGE using 4–12% Bis–Tris NuPAGE gels in MES running buffer (Thermo Fisher Scientific) following the manufacturer’s protocol. The proteins were transferred using XCell II blot module (Thermo Fisher Scientific) to a PVDF membrane (Invitrogen). Following transfer, the membranes were blocked in 5% w/v blotting-grade blocker (Bio-Rad Labs, Hercules, CA) in (TRIS)-buffered saline supplemented with 0.1% Tween-20 (Sigma, Saint Louis, MO) for 1 h. Membranes were probed with primary antibodies to H_V_1 (kind gift from Dr. Melania Capasso, Deutsches Zentrum für Neurodegenerative Erkrankungen in der Helmholtz-Gemeinschaf, Bonn, Germany), NHE-1, AKT, phosphoAKT_Ser473, Na^+^/K^+^-ATPase (Cell Signaling, Beverly, MA), and Cas9 (Takara Bio USA, Inc., Mountain View, CA) followed by incubation with an anti-rabbit secondary antibody conjugated to horseradish peroxidase (Cell Signaling, Beverly, MA). Protein bands were visualized using enhanced chemiluminescent reagent (Perkin-Elmer, Waltham, MA) and HyBlot-CL autoradiography film (Harvard Bioscience, Inc.). Densitometry was performed using ImageJ analysis software (NIH).

### Immunostaining

MDA-MB-231 cells were harvested by brief trypsinization followed by plating onto 12 mm round coverslips (thickness, #1.5) coated with poly-D-lysine. Following overnight attachment in medium, the cells were washed rapidly with warm PBS and then fixed with the addition of 4% paraformaldehyde in 200 mM phosphate buffer (pH 7.4) for 30 min. Cells were permeabilized with 0.3% Triton X-100 in phosphate-buffered saline. After Triton X-100 washout, cells were blocked overnight in buffer containing 10% goat serum (Sigma-Aldrich, St. Louis, MO). The expression of the proton channel was detected by incubation with a rabbit polyclonal primary antibody raised against human HVCN1 (gift from Dr. Melania Capasso, Deutsches Zentrum für Neurodegenerative Erkrankungen in der Helmholtz-Gemeinschaf, Bonn, Germany) used at a 1:100 dilution for 3.0 h. Following a washout period, coverslips were incubated with an Alexa-fluor 488-conjugated anti-rabbit secondary antibody. After a secondary antibody washout period, the cells were mounted in DAPI Fluoromount-G (Southern Biotech, Birmingham, AL) for nuclear co-labeling. All coverslips were evaluated with an inverted Leica microscope and confocal microscopy. Confocal images (1.0 μm) were acquired from the immunopositive cell surface membrane to the cell bottom for determination and evaluation of sections through the center of the nucleus. A plane of focus centered through the nucleus (a confocal midpoint plane positioned relative to the total nuclear thickness) and thus, deeper into the cytosol would reveal any significant membrane bound vesicular pool of HVCN1.

### Creation of luciferase expressing, HVCN1 knockdown and knockout cells

Lentiviral HVCN1 knockdown mission shRNA plasmids (SHCLNG MISSION shRNA Bacterial Clone Olig# TRCN0000165728, TRCN0000161821, TRCN0000162585) were obtained from Sigma (Cambridge, MA, USA). The lentiviral packaging and envelope vectors psPAX2 and pMD2.6 (Addgene #12260 and #12259, respectively; depositing lab Didier Trono), scramble shRNA (pLKO-vector backbone; Addgene plasmid # 1864 deposited by Dr. David M. Sabatini) [30], LentiCas-9BLAST (Addgene #52962; gift from Feng Zhang) [31]; were utilized. Luciferase (red) lentiviral vector (*Luciola italica*) (pLenti-II-CMV-Luc-IRES-GFP) was obtained from ABM (Richmond, BC, Canada). The CRISPR guide sequence (gRNA) was designed using crispr.mit.edu and the sequence of HVCN1 from the UCSC Genome Browser (NM_0322369). The gRNA guide sequence (TTAAGGCACTTCACGGTCGT) was ligated into a pLentiGuide-Puro or pLentiGuide-Neo plasmid (GenScript; Piscataway, NJ). Lentiviral supernatants for the expression of shRNAs, Cas9 plasmids, luciferase plasmids and gRNA plasmids were generated from 293FT cells using psPAX2 and pMD2G packaging and envelope vectors or 2^nd^ generation packaging mix from ABM (Richmond, BC, Canada). HEK293FT cells were grown to 40% confluence in 75 ml tissue culture flasks. Medium was removed and replaced with DMEM, 10% FBS without antibiotics. Fugene 6 (Promega, Madison, WI) or Lentifectin (ABM, Richmond, BC, Canada) was used to transfect the psPAX2, pMD2.G and expression plasmid in a ratio of 2:1:3 concentration or using the manufacturers protocol. After 48 hours the virus supernatant was collected and filtered through a 0.44 mm syringe filter and then concentrated using Lenti-X concentrator (Takara Bio USA Inc., Mountain View, CA) following the manufacturer’s protocol and resuspended in the appropriate medium. To transduce cells with virus, cells were grown in 6-well cell culture plates; the medium removed and replaced with 1 ml of the virus medium added to the wells supplemented with polybrene. After 4–24 hours of incubation the medium was replaced with fresh medium and the cells were left to recover overnight. Transformed cells were then trypsinized and replated in medium containing a selection chemical (20 μg/ml blasticidin, 2 μg/ml puromycin or 600 μg/ml neomycin). After the colony stabilized from the selection challenge, 200 μl of a cell suspension was plated at a seed density of 1 cell/ 200 μl into each well of a 96 well plate. Single cell colonies formed in the wells, were isolated and grown up to confluence in 6-well tissue culture flasks. Protein isolates were made from successful colonies and assayed for the expression of various proteins by western blot. Primers flanking the Cas9-mediated cut site were used to amplify this region for subsequent sequencing to determine the change to the sequence of the gene. Putative off-targeting sites that were predicted by crispr.mit.edu (Zhang Lab; Cambridge, MA) were also sequenced to determine if off-targeting of the Cas9 protein occurred.

### *In-vivo* measurement of xenograft growth

32 two-month-old NOD.Cg-Prkdc<scid> female mice (Jackson Labs, Sacramento, CA) were housed in groups of 6 mice under standard 12 hour light/dark cycles with food and water *ad libitum*. The number of mice required was determined using a statistical power analysis to achieve 0.80 power (at α = 0.05) to detect differences of 20% or greater (ANOVA statistical test; validated using StatMate2 [GraphPad Software, Inc., La Jolla, CA]). Based on preliminary and published data we estimated a minimum of 12 mice per experimental and control groups for these experiments. All procedures were approved by the Rush University Medical Center Institutional Animal Care and Use Committee (IACUC) in accordance with the NIH Guide for the Care and Use of Laboratory Animals. MDA-MB-231 cells transfected with luciferase gene without any genetic modifications (WT-Luc), stably transfected with Cas9 (Cas9-luc) or with HVCN1 genetically deleted (4a-luc) were resuspended at 1x10^6^ cells/100 μl in 50% DMEM/ 50% Matrigel Matrix High Concentration (HC) (Corning, Tewksbury, MA) and kept on ice. Mice were randomly assigned to groups and deeply anesthetized using 3% isoflurane gas mixed with pure oxygen and 150 μl of a cell suspension was injected into the right lower mammary fat pad (12 mice in each group; WT-Luc (control 1), Cas9-luc (control 2) and 4a-Luc (test group)). Mice were returned to their cages and allowed to recover. To analyze the bioluminescence from growing cells, mice were again anesthetized with 3% isoflurane/O_2_ and then maintained at 2% isoflurane/O_2_. Mice were then injected intraperitoneally with a 200 μl mixture of saline with 15 mg/ml of luminol at a dose of 200 mg/kg (GoldBio, St Louis, MO, USA) and the tumors imaged by placing the mice under the CCD camera of an IVIS Lumina *in vivo* imaging system (Caliper Life Sciences; Hopkinton, MA) and acquiring images every 30 s for up to 45 min. The mice were retained in position under isoflurane anesthesia (1.5%- 2.0%) within the imaging system compartment for the duration of imaging. Luminescent images from peak levels (~20–30 min after injection) were analyzed using Living Image 4.1 software (Perkin Elmer) and ImageJ, calculating the integral and area of the luminescent signal above background that was recorded for each tumor. Mouse well-being and tumor sizes were recorded in the mornings twice a week in the procedure rooms in the Rush University CRC animal facility. Experiments continued until tumors reached 2 cm in size, or 10% of body mass, or a moribund condition resulted, whichever came first. The experiment was concluded after 5 weeks when 5 mice in the WT-LUC and CAS9-LUC met the requirements for euthanasia.

The mice were euthanized by CO_2_ asphyxia and the tumors were resected and weighed immediately. Tumors were then frozen in RNA stabilization solution (Sigma-Aldrich, St. Louis, MO) and stored at -80°C for subsequent use. Tumors were fixed in formalin and embedded in paraffin wax, sectioned and stained with hematoxylin/eosin and immunostained for Ki-67, the nuclear protein marker for cellular proliferation. Tumor sections were photographed at x100 and x40 magnification from three fields of view in well stained areas. The level of staining was assessed by image deconvolution in ImageJ to assess the number of positively stained cells within each image. Ki67 positive cells were counted in each X100 field and compared across the tumor types. Statistical comparison of tumor weight and Ki67 staining was performed by one-way ANOVA with significance assumed *p*<0.05.

### Real time live-cell extracellular acidification rate measurement (ECAR)

WT MDA-MB-231 cells, Cas9 expressing cells, SRC, shRNA knockdowns, and 2 knockout clones (4a and 5f_2_) cells grown in T75 tissue culture flasks were harvested at 70–90% confluence and resuspended in DMEM 10% FBS, penicillin/streptomycin at 100,000 cells/ml. 200 μl of this cell suspension was plated into a pre-calibrated Seahorse cartridge (Agilent Technologies, Santa Clara, CA) and allowed to attach overnight in 1% serum. Medium was exchanged with Seahorse assay medium and the cells incubated in the absence of glucose for one hour. The cells were then analyzed per manufacturer instructions for real time extracellular acidification rate (ECAR) (Agilent Technologies). Briefly, the medium was removed and replaced with Seahorse specific assay medium until they were analyzed in the Seahorse XF Analyzer for live-cell metabolic assays. The Seahorse overlay had solutions of 1 M glucose, 10 mM oligomycin, and 500 mM deoxyglucose (final concentrations of 10 mM glucose, 100 μM oligomycin, and 5 mM deoxyglucose). The experiment was designed so that baseline recordings before addition of glucose were every 4 min, followed by readings every 8 min after glucose addition. Oligomycin was added 16 min after glucose addition and 2-deoxyglucose was added 16 min after the deoxyglucose. The units for ECAR are mpH/min (rate of change for extracellular well acidification) as calculated by the Agilent Seahorse XF analyzer software.

### Amplex Red assay of cellular hydrogen peroxide levels

Cells were grown as for the Seahorse ECAR measurement above but were harvested on the day of the experiment and washed twice in ice cold Ringer’s solution without EGTA by centrifugation at 400 x g for 5 min. Cells were then resuspended in Ringer’s solution with 25 mM glucose added and kept on ice until the start of the experiment. A stock solution of 100 μM Amplex red in Ringer’s solution was prepared and 50 μl of the reaction mixture was added to each well of a 96 well plate. 200,000 cells in Ringer’s suspension in 150 μl was added to each well and the release of H_2_O_2_ from the cells was recorded over the course of an hour in a Perkin Elmer Victor3V cell plate reader set at 37°C with an excitation wavelength at 540 nM and emission wavelength at 595 nM. A standard curve of H_2_O_2_ was performed with each experiment with concentrations of H_2_O_2_ running from 100 nM to 10 μM in order to calibrate the recording.

### Reverse Phase Protein Array (RPPA)

WT MDA-MB-231 cells, Cas9 expressing cells and 3 H_v_1 KO clones (4a, 5f2 and 1fb) were seeded in T-75 culture dishes and allowed to adhere for 24 hours in DMEM with 0.5% FBS. The medium was replaced with assay complete DMEM and the cells were cultured for a further 24 hours. Cells were harvested by brief trypsinization followed by centrifugation at 400 x g for 5 minutes. Cell pellets were then flash frozen in liquid nitrogen and sent to the RPPA core facility at MD Anderson Cancer Center, Houston, TX. At the core facility the cell pellets were lysed and serially diluted 2-fold 5 times and arrayed on nitrocellulose slides. The samples were probed with 300 antibodies verified by the MD Anderson core by the tyramide-based signal amplification approach and visualized by DAB colorimetric reaction. The images were scanned and spot density was quantified by Array-Pro analyzer. The data points were normalized to protein loading, transformed to Log2 values, median centered and formatted for the heatmap figure.

### RT-PCR for NOX mRNA in MDA-MD-231 cells

Cells were harvested by trypsinization (0.5%) for 3 minutes and centrifuged at 400 x g for 5 minutes. Total RNA was isolated using the RNeasy kit (Qiagen, Valencia, CA). Amplicons were generated using SuperScript^™^ One-Step RT-PCR System with Platinum^™^ Taq DNA Polymerase

(Thermo Fisher Scientific) following the manufactures instructions.

cDNA was probed using primers designed in house and ordered from Integrated DNA technologies (Coralville, Iowa). Primer design was as follows: -

H_V_1: Forward CTTGACTTCAGGGGCATGTT, Reverse TCTTCAGGTCCAGGATGAGC;

NOX1: Forward TGTAGGCGCCCTAAGTTTGA, Reverse ATCACAACCTTCTGCTGGGA; NOX2: Forward GCCAGTCTGTCGAAATCTGC, Reverse ATCATCCATGCCACCATTTT; NOX3: Forward ACAGACCCCACTGAAGCTGA, Reverse CCGTGTTTCCAGGGAGAGTA; NOX4: Forward CTGCTGACGTTGCATGTTTC, Reverse AACCAACGGAAGGACTGGA; NOX5: Forward TTTCGAGTGGTTTGTGAGCC, Reverse CCTTCATGTCATTCTTGCCC; DUOX1: Forward AGTTCCTGGACATCCTGGTG, Reverse GAACATGGACTCCACCACCT; DUOX2: Forward GGCTCCCCAGAGGATAAGTC, Reverse GTCAGCTCCTCCTTGTCCTG.

T_m_ values were 52.5°C and thermocycling was performed for 35 cycles. Products were run on a 2% agarose gel in 45 mM Tris-Borate buffer, 1 mM EGTA with 1 μg/ml ethidium bromide for visualization.

### Scratch test assay for cell migration

24 well culture plates were coated with fibronectin by addition of 500 μl of 5 μg/ml fibronectin in PBS for 1 h at room temperature. The excess solution was aspirated and the wells washed with PBS. The underside of each well was marked in the center with a scorer to identify the intended wound area. MDA-MB-231 cells (Cas9 expressers, shRNA, KO) were plated at 2x10^5^ cells per well in 24-well plates (Thermo Fisher) and allowed to adhere and form a monolayer overnight. A wound was made in the monolayer using a 100 μl pipette tip to draw a line through the cells in a single fluid motion. The media was removed by aspiration and each well was washed carefully with 1 ml of PBS. 500 μl of DMEM with 0.5% FBS was added to each well. Pictures were taken at *t* = 0 using a Cannon EOS Rebel T3i attached to the side arm of a Nikon eclipse TE200. The plates were incubated at 37°C in 5% CO_2_ for 24 hours when pictures were taken again using the underside scratches to determine location of the previous picture. Images were converted to Tiff files and opened in ImageJ for analysis of the area remaining after wounding. One-way ANOVA was used to determine significance between the different test groups.

### RNA-sequence cell preparation, data acquisition, quality control, and processing

Cells were prepared in the same manner as for RPPA analysis and the samples sent to Genewiz (South Plainfield, NJ) for RNA-sequence (seq) analysis. RNA-seq read summarizations were aligned to the Ensembl top-level assembly with STAR version 2.0.4b. Gene counts were derived from the number of uniquely aligned unambiguous reads by Subread:featureCount version 1.4.5 program. Transcript counts were produced by Sailfish version 0.6.3. Sequencing performance was assessed for total number of aligned reads, total number of uniquely aligned reads, genes and transcripts detected, ribosomal fraction known junction saturation and read distribution over known gene models with RSeQC version 2.3.

All gene-level and transcript counts were then imported into the R/Bioconductor package EdgeR and TMM normalization size factors were calculated to adjust for samples for differences in library size. Ribosomal features as well as any feature not expressed in at least the smallest condition size minus one sample were excluded from further analysis and TMM size factors were recalculated to create effective TMM size factors. The TMM size factors and the matrix of counts were then imported into R/Bioconductor package Limma and weighted likelihoods based on the observed mean-variance relationship of every gene/transcript and sample were then calculated for all samples with the voomWithQualityWeights function. Performance of the samples was assessed with a Spearman correlation matrix and multi-dimensional scaling plots. Gene/transcript performance was assessed with plots of residual standard deviation of every gene to their average log-count with a robustly fitted trend line of the residuals. Generalized linear models were then created to test for gene/transcript level differential expression. Differentially expressed genes and transcripts were then filtered for mean 2 fold difference between WT + Cas9 and 4a + 5F_2_.

## Results

### Electrophysiological measurement of native H_V_1 in MDA-MB-231 cells

**Fig 1:**
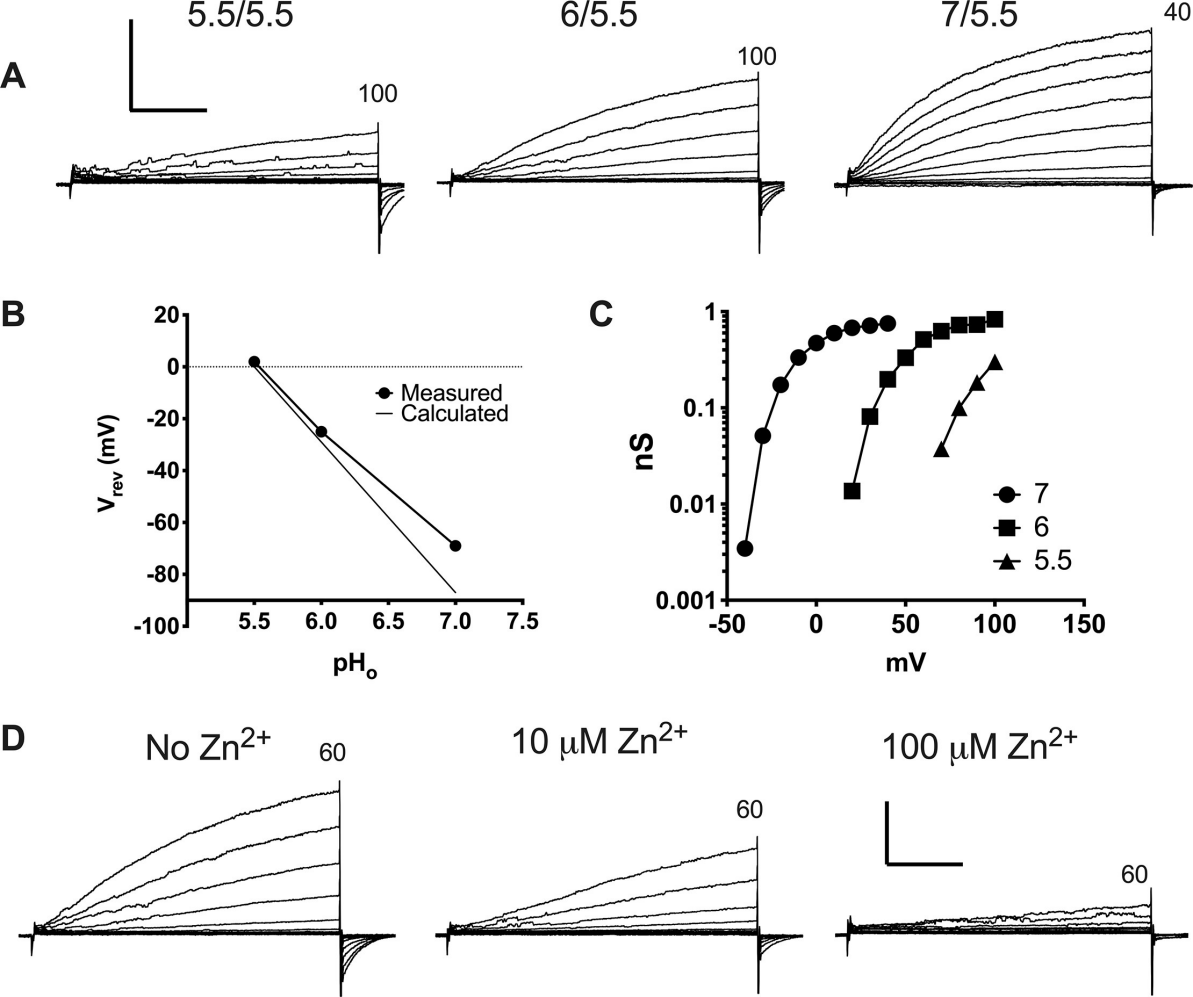
Proton current recorded in whole cell patch clamp configuration in an MDA-MD-231 breast cancer cell. (**A**) Three families of currents at different pH_o_ all with a pH_i_ of 5.5 (labeled pH_o_/pH_i_). Voltage steps are every 10 mV, up to the numbers listed at the top of each family (in mV). The membrane was held at -40 mV (pH 6/5.5 and 5.5/5.5) or -60 mV (pH 7.5/5.5), and pulses were applied with an interval of 20s. The scale bar units are 100 pA and 2 s. (**B**) The reversal potential (*V*_rev_) of a representative cell measured at each pH_o_ compared to the Nernst potential for protons, which assumes perfect selectivity for H^+^. (**C**) Proton conductance (*g*_H_) voltage curves derived from the data in **A** using *V*_rev_ measured in each solution. (**D)** Three families of currents in a cell at pH 6/5.5 with different concentrations of Zn^2+^ as labelled. Voltage steps are 10 mV apart up to 60 mV. The scale bar units are 50 pA and 2 s.

The studies by Wang et al. [21, 22] showed expression of H_V_1 by western blotting and PCR but lacked functional data showing proton currents. We examined MDA-MB-231 cells by patch clamp technique and recorded *bona fide* voltage-gated proton channels confirmed by their kinetics, pH dependence, voltage dependence, proton selectivity, and sensitivity to Zn^2+^ (Fig 1). We were not always able to record definitive H_v_1 current in these cells. Some cells exhibited an extraneous conductance similar to that reported previously in COS-7 cells [32]. In five out of nineteen cells proton currents were absent despite little obscuring noise. The cells with proton currents varied quite considerably in size with capacitance ranging 7–92 pF with a mean value of 35 ± 25 (mean ± SEM, *n* = 14). In 4 cells with proton current the average maximum proton conductance, *g*_H_, at pH 5.5/5.5 was 37.4 pS/pF. Fig 1A shows representative proton current families recorded in an MDA-MB-231 cell at 3 different pH gradients; pH 5.5/5.5, 6/5.5 and 7/5.5 (pH_o_/pH_i_). The current traces show clear pH dependent gating with currents becoming larger and activation occurring at more negative voltages as the pH gradient increases from symmetrical pH to a 1.5-unit outward pH gradient. The current amplitude at 60 mV averaged 67.7 ± 38 pA (*n* = 6) at pH 7/5.5 and 16.7 ± 18 pA (*n* = 9) at pH 5.5/5.5. The reversal potential of the currents in this cell is plotted in Fig 1B for comparison with *E*_H_ values calculated with the Nernst equation. The small deviation from the Nernst potential most likely reflects incomplete control over pH_i_ when there is a large pH gradient. In four other cells, currents reversed at -69 ± 14 mV at pH 7/5.5 and at 8 ± 6 mV at pH 5.5/5.5. The proximity of the measured reversal potential with the Nernst potential confirms that these channels are highly selective for protons.

Fig 1C shows conductance voltage curves for the representative cell. As observed in all species with H_V_1 currents, the *g*_H_-V relationship shifts about 40 mV per unit decrease in the pH gradient (pH_o_—pH_i_) [7, 9]. Measured at 60 mV, the time constant for turn-on of current (*τ*_act_) was 3.2 ± 1.3 s at pH 7/5.5 and 6.3 ± 3.2 s at pH 5.5/5.5 (*n* = 4). This slow activation is characteristic of human H_V_1 [1, 33–35] and more generally, mammalian H_V_1[36–38]. The currents were sensitive to Zn^2+^ as is characteristic of proton currents [6, 39, 40]. Measured at pH_o_ 7, 1 μM Zn^2+^ reduced currents and slowed the current turn-on in 3 cells. Fig 1D illustrates that increasing Zn^2+^ progressively attenuates the current, slows activation, and shifts the *g*_H_*-V* relationship positively, characteristic divalent metal cation effects on H_V_1 in many species [40, 41].

### *In-situ* staining of H_V_1 in cells

**Fig 2:**
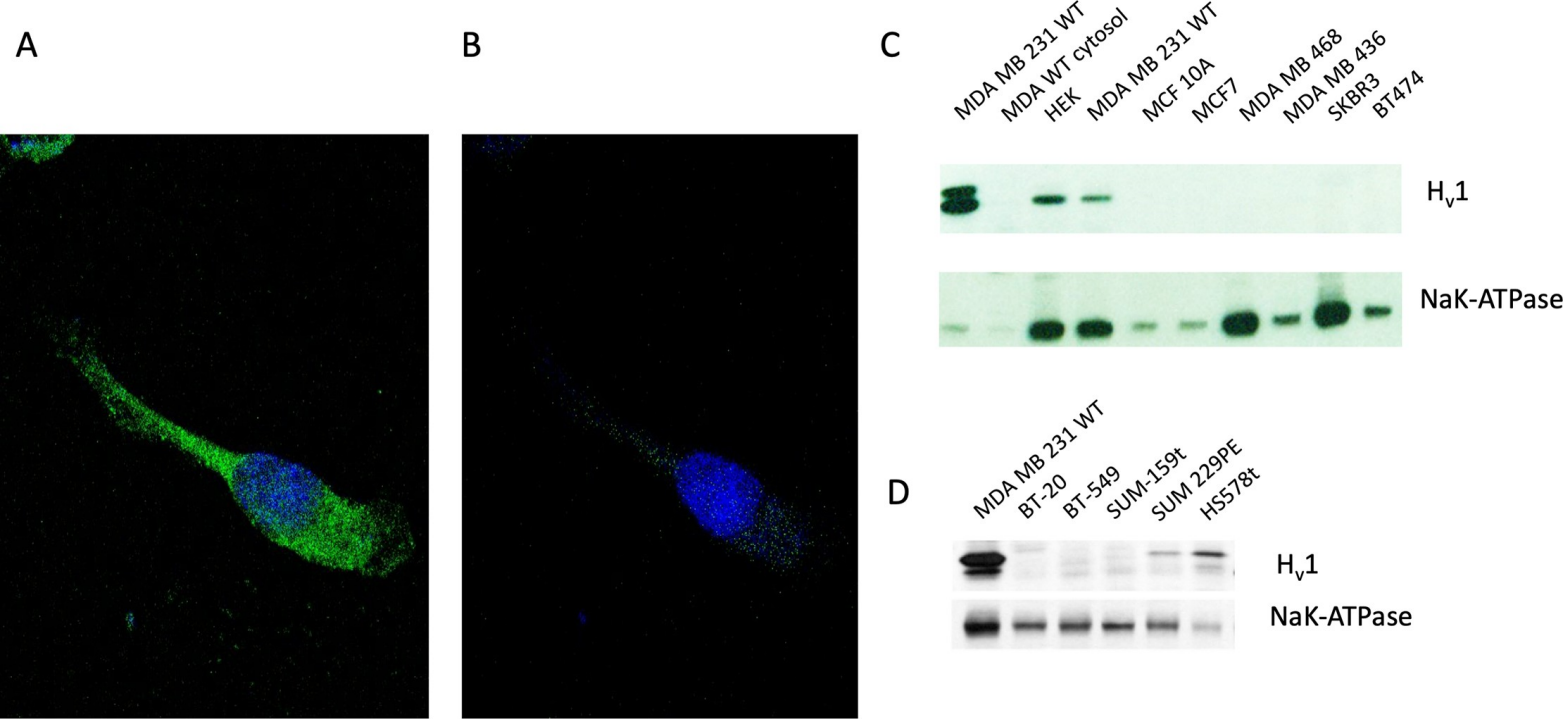
H_V_1 protein is present in the plasma membrane of MDA-MB-231 cells and in other triple-negative breast cancer cell lines. (**A**) An MDA-MB-231 cell permeabilized with Triton X-100 and stained with an antibody specific for H_V_1 with the image focused on the plane of the membrane surface. (**B)** Shows the fluorescence of the same cell but with the focus centered through the nucleus (a confocal midpoint plane positioned relative to the total nuclear thickness) and thus, deeper into the cytosol. The nucleus was stained with DAPI and is visible in both **A** and **B**. (**C**) Western blotting of membrane fractions from a number of different breast cancer cell lines. Membrane fractions were stained with antibodies specific to H_V_1 and Na^+^/K^+^-ATPase as a membrane fraction loading control. The top pair of blots examines membranes from a number of cells representative of the major types of breast cancer, i.e., Luminal A, Luminal B, Her2 and triple negative. (**D**) Western blots from 6 different triple negative breast cancer cells.

Immunostaining of fixed permeabilized cells (Fig 2A and 2B) revealed significant staining for H_V_1 in the plane of the plasma membrane as visualized with fluorophore-conjugated secondary antibodies (AlexaFluor-488) and confocal microscopy. However, little or no staining (relative to negative control; minus primary antibody -not shown) was evident in the cytosolic regions of the cell when the cell was permeabilized to allow antibody access to the cell interior (Fig 2B), suggesting that the channel is expressed exclusively in the plasma membrane of the MDA-MB-231 cancer cell.

In order to find the optimal model system to study the effect of H_V_1 expression on cell function we screened 12 different breast cancer cell lines for H_V_1 [42] using western blot techniques. Cell types were chosen from a variety of different molecular classifications including luminal A, luminal B, basal-like, HER2-positive and normal subgroups. We homogenized and fractionated cell lysates to examine the membrane fractions for the presence of H_V_1 protein (Fig 2C and 2D). Three cell lines expressed H_v_1 distinctly when comparing equivalent membrane protein amounts: MDA-MB-231, Hs578t, and Sum229PE. Of these 3 cell lines the highest expression levels were in MDA-MB-231 cells. Attempts to record H_V_1 by patch clamp from HS-578t and Sum229PE cells were not successful. The cell lines that expressed H_V_1 are all classified as basal or triple negative breast cancer cell lines, because they do not express estrogen receptors, progesterone receptors, or the HER-2 protein. We chose to move forward with the MDA-MB-231 cells as a model because their H_V_1 expression was highest among the dozen cell lines tested.

Intriguingly, in the cell lines expressing H_V_1 protein, both the full-length protein (273 amino acids) was detected, but also often a shorter form. This is most prominent in Fig 2C, top panel, where a HEK cell with overexpressed H_v_1 protein shows a prominent second band. A short form of human H_V_1 was reported previously in a number of malignant B cell lines, in particular chronic lymphocytic leukemia [20]. This protein lacked the first 20 amino acids due to a secondary initiation site at Met^21^. In Fig 2D H_v_1 short form is also visible in MDA-MB-231 and Hs-578T cells. The presence of the short form of H_v_1 in western blots was variable, possibly due to the concentration of protein loading and photographic film exposure times. Often, we only saw one band (see Fig 2C) which we assume is the long form as it is the most dominant form in most cells.

We used the blotting of Na^+^/K^+^-ATPase to show the validity of the membrane preparation as it is a ubiquitously expressed membrane protein. We were able to detect the presence of the Na^+^/K^+^-ATPase in every sample. Because its expression varied in each different cell type it was not used to normalize H_v_1 expression.

### The effect of shRNA knock-down (KD) of H_V_1 on the migration and H_2_O_2_ production of MDA-MB-231 cells

**Fig 3:**
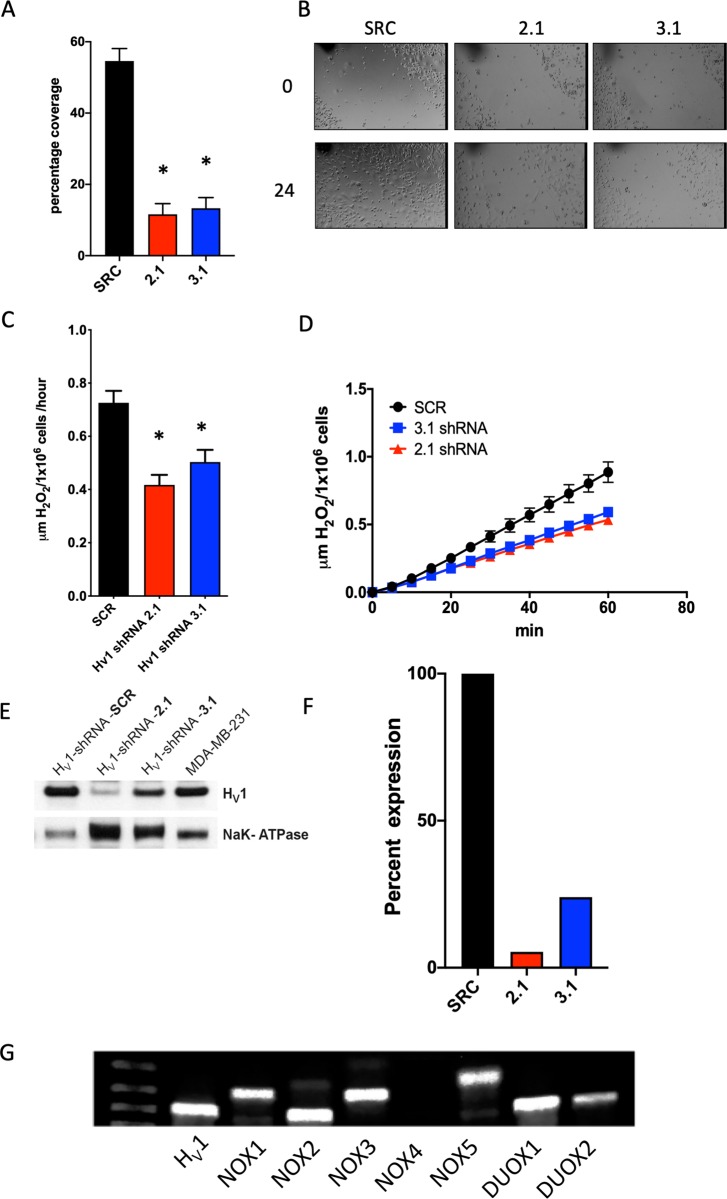
KD of H_V_1 slows cell migration in a scratch assay and inhibits H_2_O_2_ release. (**A**) Quantitative analysis of 3 wound healing assays performed in triplicate as illustrated by the example in **B**. The graph shows the percentage coverage of each wound by cells after 24 h, analyzed using ImageJ. Differences are significant as determined by a normal one-way ANOVA with Bonferroni's post-test (*p*<0.0001). (**B**) Images of wells 24 hours apart after wounding with a 200 μl pipette. The black marks denote position. MDA-MB-231 cells were treated with virus containing the sequence for SCR (control), or 2.1 or 3.1 shRNA for H_V_1 knockdown. Cells were kept in 1% FBS with high glucose. (**C**) The release of H_2_O_2_ from MDA-MB-231 cells in one hour. The data summarize 4 experiments performed in triplicate. 2.1 and 3.1 are significantly different from SRC as determined by a one-way ANOVA with Bonferroni's post-test (*p*<0.0015). (**D**) A representative experiment depicting the release of H_2_O_2_ over one hour. (**E**) A Western blot showing the inhibition of H_V_1 expression in MDA-MB-231 cells by shRNA 2.1 and 3.1. Na^+^/K^+^-ATPase was used as a membrane fraction loading control. (**F**) A graph showing the expression of H_V_1 normalized to the expression of Na^+^/K^+^-ATPase as determined by densitometry for the data shown in F. (**G**) PCR amplification of products specific to the 7 members of the NOX family of membrane proteins.

Wang et al. [21] described a reduction in migration kinetics when using siRNA in the MDA-MB-231 cells to reduce the expression of H_V_1. We used a similar approach but with shRNA knock-down of H_V_1 in MDA-MB-231 cells. We tried several Mission shRNA sequences (Sigma-Aldrich, St Louis, MO) packaged in lentiviral vectors to transfect MDA-MB-231 cells in order to reduce the expression of H_V_1 and found 2 shRNA sequences that were effective in significantly knocking down the expression of H_V_1 (Fig 3E). The successful sequences used, labeled 2.1 and 3.1 cells, reduced the expression of H_V_1 in MDA-MB-231 cells to 5.6 ± 2% and 24.1 ± 4% of the SRC control expression (mean ± SEM, *n* = 3), respectively. We replicated the migration assay by growing cells to confluence in 6-well plates, serum starving the cells for 24 h and then wounding the monolayer with a sterile pipette tip. The cells were incubated in 0.5% serum medium to examine motility but not growth. Under these conditions the WT cells recovered 54.6 ± 7% (*n* = 4) of the wound in 24 hours whereas the cells transfected with the 2.1 and 3.1 shRNA constructs showed greatly reduced wound recovery of 11.6 ± 3% (*n* = 4) and 13.3 ± 4% (*n* = 4), respectively (Fig 3A and 3B).

H_V_1 is known to be closely associated with the activity of NADPH oxidases (NOX) in a number of cell types including neutrophils, eosinophils, B lymphocytes, and monocytes [10–14, 43–45]. Therefore, we tested the hypothesis that inhibiting the expression of H_V_1 could result in a decrease in the production of superoxide, as has been demonstrated in numerous cell types. We measured the release of H_2_O_2_ from cells treated with shRNA for H_V_1 or the scrambled control, SCR for an hour using Amplex red reagent. Cells transfected with shRNA specific to HVCN1 showed a significant reduction compared to SRC transfected control. Control cells released 0.72 ± 0.04 μmols H_2_O_2_/1x10^6^ cells/hour (*n* = 6) compared to 0.42 ± 0.03 μmols H_2_O_2_/1x10^6^ cells/hour (*n* = 4, *p*<0.001) for 2.1 shRNA and 0.50 ± 0.5 μmols H_2_O_2_/1x10^6^ cells/hour (*n* = 4, *p*<0.01) for 3.1 shRNA. PCR analysis shows that MDA-MB-231 cells express the NADPH oxidase molecules NOX 1, 2, 3, 5 and both Dual oxidases (DUOX) isoforms (Fig 3F). The wealth of NOX isoforms present provides a basis for H_2_O_2_ production. We did not pursue which contributed to the observed H_2_O_2_ production.

### Creation of HVCN1 knock-out (KO) MDA-MB-231 cells via CRISPR gene editing

**Fig 4:**
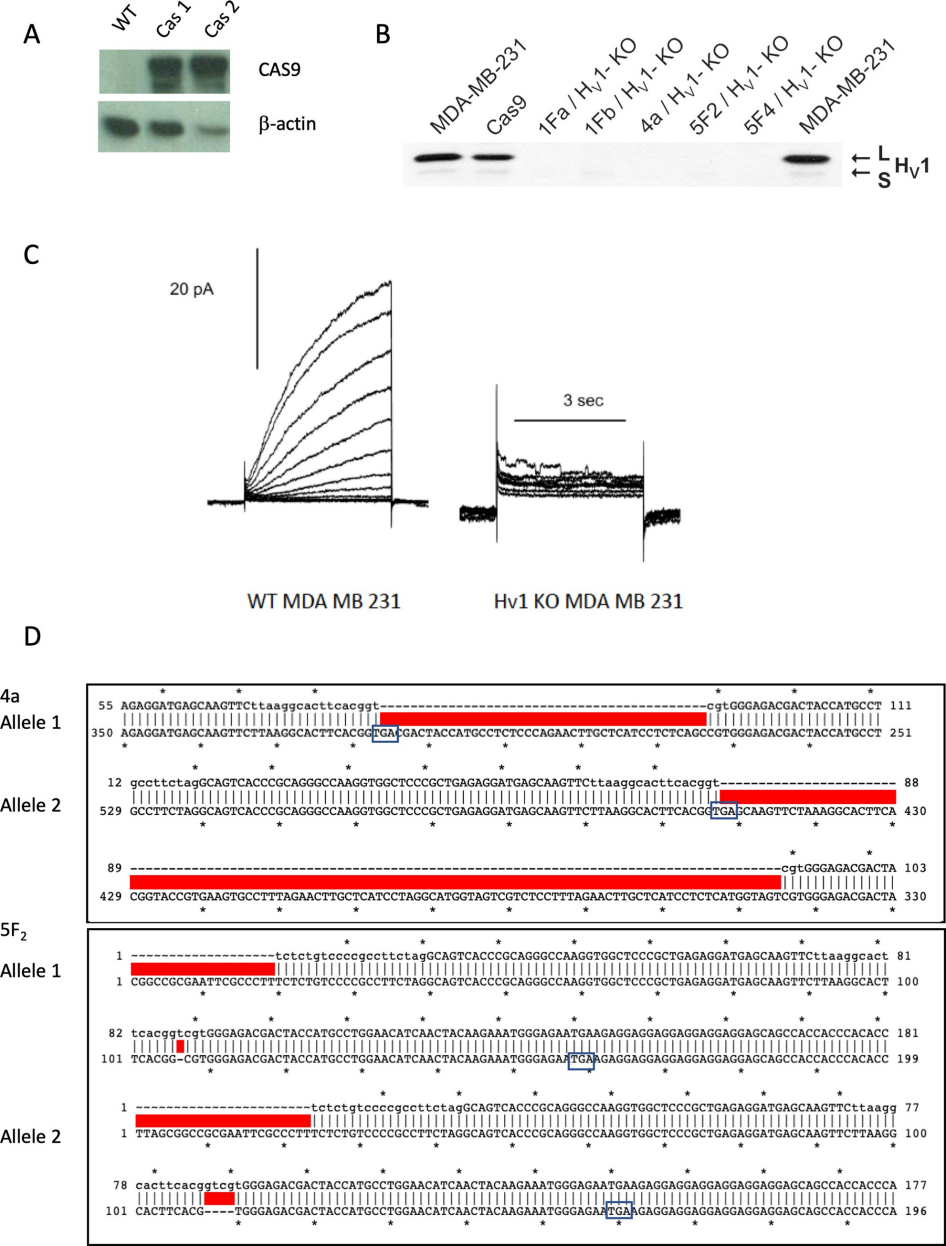
Generation of KO cells using Cas9 and CRISPR. (**A**) Western blots specific to Cas9 showing the successful transduction of Cas9 protein in MDA-MB-231 cells. (**B**) A comparison of H_V_1 expression by western blot in WT MDA-MB-231 cells and in 5 H_V_1 KO clones. (**C**) Comparison of current in WT MDA-MB-231 cells compared to a cell from H_V_1 KO MDA-MB-231 cells (4a). (**D**) The documentation of the cut site of Cas9 on the 2 alleles for the HVCN1 gene in 4a (top) and 5f2 clones (bottom). Both alleles in the 4a clone were extended by nonhomologous eníd joining, the top allele by 43 nucleotides and the bottom by 83. In the 5f_2_ clone one allele had a 4 base pair deletion and one allele had a single nucleotide deletion. In all cases a stop codon was created by frame shifts caused by the deletion or insert.

The shRNA experiments generally corroborated previously published studies. However, some studies have shown that shRNA can have off target effects not seen with KO [46]. We therefore deleted the functional HVCN1 gene altogether to examine how MDA-MB-231 cells function in its absence. To this end, we stably transfected Cas9 into MDA-MB-231 cells (Fig 4A) before we targeted exon 2 of the H_V_1 sequence using the CRISPR guide sequence TTAAGGCACTTCACGGTCGT transfected into cells using a lentiviral vector. This approach targets both the full length and short isoforms of hH_V_1 [20]. We isolated several clones generated by this approach (Fig 4B). We did not detect expression of H_V_1 by western blotting equivalent amounts of membrane protein (Fig 4B) or the electrophysiological function of H_V_1 by the patch clamp technique (Fig 4C shows clone 4a). Furthermore, sequence analysis of this clone showed that gene editing resulted in a 43-base insertion into one allele and an 83-base insertion into the second allele at the cut site for clone 4a. In clone 5f_2_, we observed in both cases a premature stop codon occurred shortly downstream of the cut site (Fig 4D).

### The effect of H_V_1 KO on migration and H_2_O_2_ production of MDA-MB-231 cells

**Fig 5:**
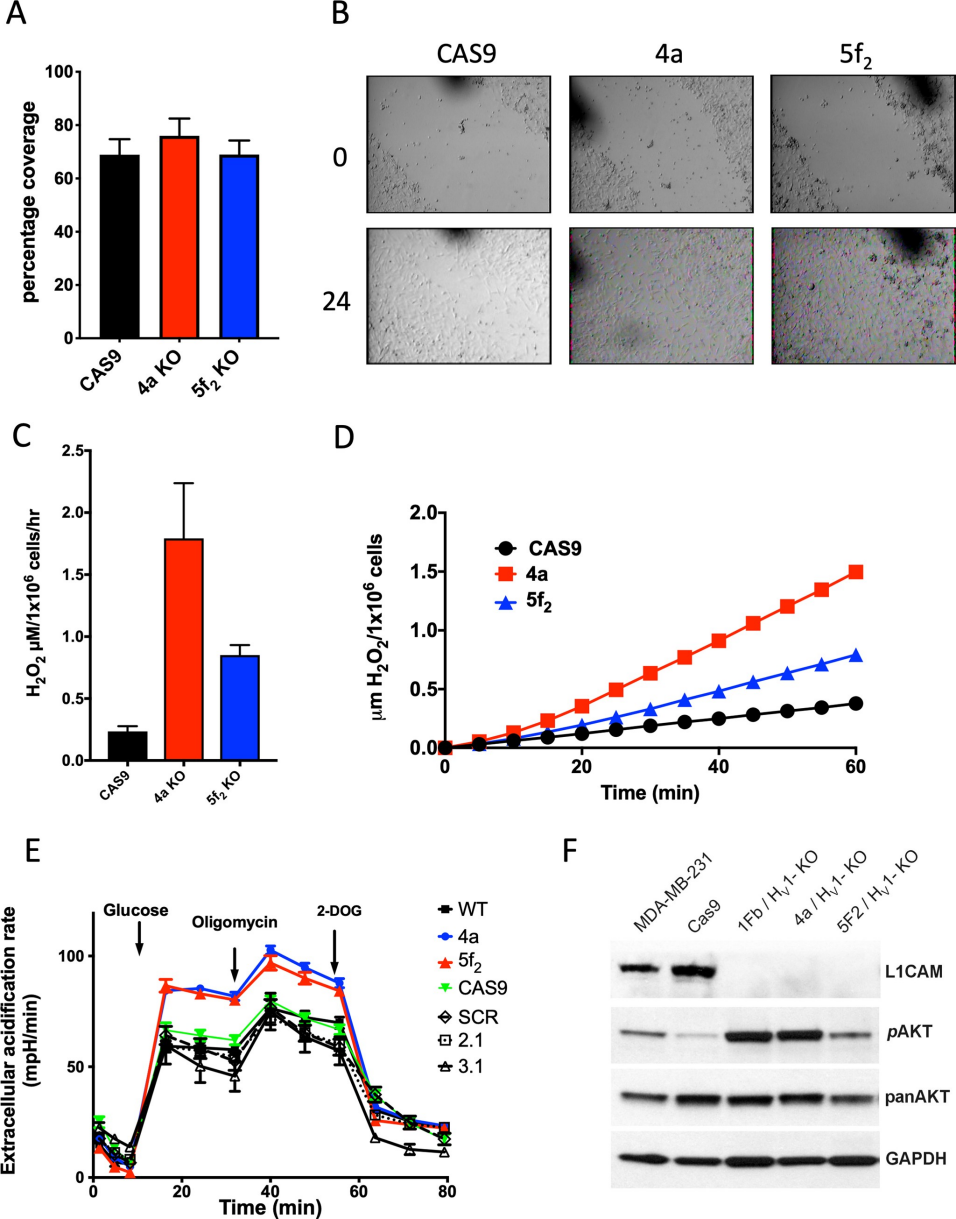
Effects of H_V_1 KO on wound healing, H_2_O_2_ production, metabolism, and bioactive molecules. (**A**) The quantitative analysis of the wound healing assay illustrated in **B**. The chart shows the percentage coverage of each wound by cells after 24 hr. Analysis was performed using ImageJ and the chart shows data from 3 experiments performed in triplicate. Data are not significantly different. (**B**) Images of wells 24 hours apart after wounding with a 200 μl pipette. The black marks are scratch marks to denote position. MDA-MB-231 cells treated with virus containing the sequence for Cas9, 4a, or 5f_2_ for H_V_1 knockout. Cells were kept in 1% FBS but with high glucose. (**C**) The release of H_2_O_2_ from MDA-MB-231 cells. The data is from 4 experiments performed in triplicate. 4a is significantly different from Cas9 as determined by a one-way ANOVA with Bonferroni's post-test *p*<0.05. (**D**) A representative experiment illustrating the release of H_2_O_2_ over one hour. (**E**) The extracellular acidification rate (ECAR) of H_V_1 KO in 2 knockout clones compared to WT MDA-MB-231, Cas9, SRC, 2.1 and 3.1 knockdowns. Extracellular acidification was increased by adding 25 mM glucose after an hour of starvation. 10 μM oligomycin was used to inhibit mitochondria and enhance glycolysis. 2-deoxyglucose (5 mM) inhibits glycolysis to reveal the contribution of glycolysis to overall extracellular acidification. Data are the mean ± SEM from 5 experiments performed in at least sextuplet. (**F**) Western blot total protein extracts from MDA-MB-231 cells showing the different levels of protein expression or protein phosphorylation between WT and Cas9 expressing MDA-MB-231 cells and 3 different H_V_1 KO clones (1fb, 4a and 5f2).

We compared the effect of KO of H_V_1 to our results with KD cells by performing the same migration experiments on the 4a and 5f_2_ clones using the KO cells, comparing the responses to Cas9 transfected cells. The Cas9 cells were chosen as the control because they were manipulated in the same fashion, having been transfected with Cas9 and then clonally isolated before being transfected with null plasmid, whereas the knockouts were transfected with a guide sequence to the H_V_1 gene. The migration of the cells in low serum medium as measured by the percentage closure of a wound over 24 hours was 68.9 ± 5.8% for the Cas9 control, 76.0 ± 6.4% for 4a cells and 68.9 ± 5.2% for the 5f2 cells (Fig 5A). None of these measurements were significantly different from each other as measured by ANOVA with Bonferroni multiple comparison posttest. When we measured the release of H_2_O_2_ from the different cell types (Fig 5C & 5D) we found that the KO cells produced more H_2_O_2_ than the Cas9-only expressing cells. The release of H_2_O_2_ from Cas9 cells (Fig 5C) was 0.23 ± 0.04 μM H_2_O_2_/1x10^6^ cells/hour (*n* = 10), while the 4a cells released 1.52 ± 0.17 μM H_2_O_2_/1x10^6^ cells/hour (*n* = 10), and the 5f_2_ cells released 0.85 ± 0.08 μM H_2_O_2_/1x10^6^ cells/hour (*n* = 10). The release of H_2_O_2_ from the 4a cells was significantly different from the release of H_2_O_2_ from both the Cas9 cells and the 5f_2_. This result seems to contradict the response observed for shRNA treated cells, although it should be noted that the Cas9 transfected cells (used as the control for KO cells) released much less H_2_O_2_ than did SCR control MDA-MB-231 cells (Fig 3C).

To explore the possibility that metabolism was altered, we analyzed the extracellular acidification rate (ECAR) of the various MDA-MB-231 cell types created. Fig 4E shows the result of starving the different cell constructs (WT, 4a, 5f_2_, Cas9, SCR, 2.1, 3.1) of glucose and then reinstating glucose and analyzing the ECAR. Under these conditions both of the H_V_1 KO clones showed a significant increase in ECAR with 4a and 5f2 cells increasing to 84.3 ± 1.7 mpH/min and 86.6 ± 2.9 mpH/min (*n* = 5), respectively, compared to 59.3 ± 2.7, 66.4 ± 3.3, 64.1 ± 4.1, 59.2 ± 2.6 and 59.8 ± 8.7 mpH/min for WT, Cas9, SRC, 2.1 and 3.1. The KO cells were significantly different from the other cells at *p*<0.001 after addition of glucose. Treatment of cells with oligomycin inhibits the mitochondrial electron transport chain and causes metabolism to shift completely to glycolysis. Treating the cells with oligomycin increased the ECAR values in all cells, with the H_V_1 KO cells remaining significantly higher than the other cell types (*p*< 0.001) showing that they had a greater glycolytic reserve compared to non-KO cells.

Consistent with the enhanced ECAR values, we found an increase in phosphorylated AKT (Ser473) (Fig 5F) which is known to be a driver of glycolytic metabolism [47]. Additionally, upon assaying several different protein markers that were suggested from a reverse phase protein array (RPPA) screen, we found a dramatic reduction in the expression of the membrane adhesion molecule L1CAM to below detection levels in three different knockout clones (Fig 5F).

### Assessing *in-vivo* tumor growth of WT versus H_V_1 KO MDA-MB-231 WT cells

**Fig 6:**
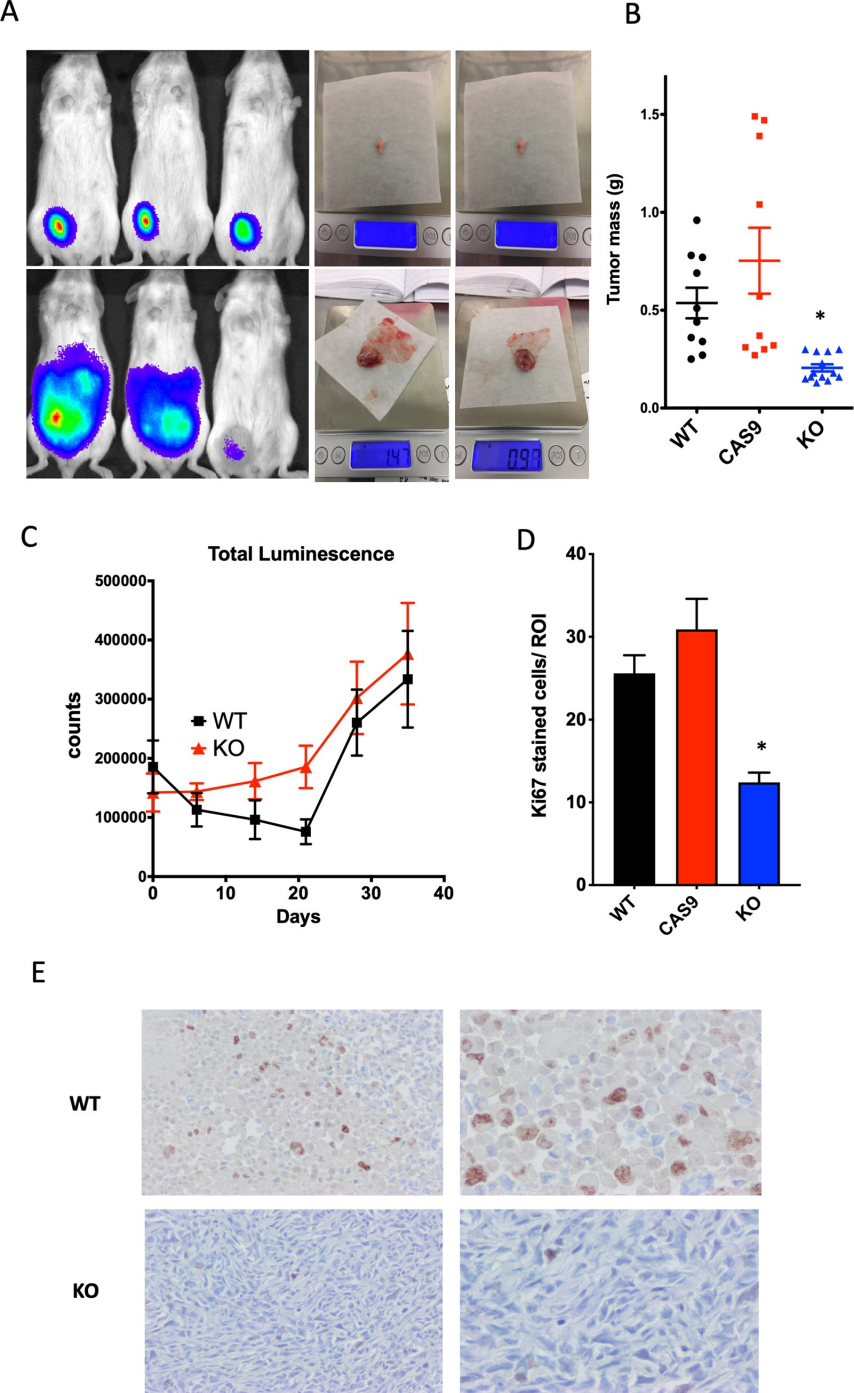
H_V_1 KO attenuates the growth of breast cancer cell-derived tumors in mice. (**A**) Three representative mice with tumors in their mammary fat pad at the end of 35 days following inoculation with MDA-MB-231 cell clones. Images illustrate the luminol-based bioluminescent visualization of the *in vivo* tumors after peak levels (~20–30 min post-luminol injection) of luminescence are achieved showing a general gradient of intensity from tumor center to surround (red-center, most intense luminescence; blue-surround, least intense). Top left panel shows mice with tumors from H_V_1 KO 4a cells and the lower left panel shows tumors from wild type cells. The right panels show 2 representative tumors from each group shortly after dissection. (**B**) A dot plot graph that categorizes the different weights of the various tumors that were resected from the mice in each group. The mean ± SEM is plotted in each case. There were significant differences between the 4a group and the WT and Cas9 group (*p*<0.05). (**C**) A graph comparing the integrated luminescence intensities of tumors as measured every seven days by injection of 200 mg/kg luminol into each mouse and a post-injection time allowance for the luminescent intensity to plateau. The luminescence counts are normalized to the relative expression determined by luminescence intensity measured in 1x10^6^ cells *in vitro*. (**D**) Number of Ki67 cells stained in each X 100 region of interest (ROI) for the different tumor types (WT, CAS9 and KO). The data are mean ± SEM significance was determined by one-way ANOVA with Bonferroni's post-test (p <0.001). (**E**) Bright-field microscopic images of stained tissue sections at X 40 (left) and X 100 (right) of 2 representative tumors taken from the WT group (top) and 4a group (bottom) that were immunostained for Ki67 (brown staining); hematoxylin and eosin background staining.

We next assessed whether the deletion of the functional H_V_1 gene would affect an *in-vivo* model of tumor growth. Previous studies [22] had shown that knockdown of the *HVCN1* gene resulted in slower tumor growth in a mouse model. Because we observed incongruous responses of knockdown and knockout cell models, we examined the growth of wild-type MDA-MB-231 cells compared to Cas9 expressing MDA-MB-231 cells and H_V_1 KO MDA-MB-231 cells. To monitor the growth of the tumors visually as well as via physical measurements we transfected the cells with a firefly luciferase plasmid in order to detect the growth of the tumor cells via bioluminescence without resorting to surgical methods. The cells were injected into the mammary fat pad of NSG/SCID mice and the mice were allowed free access to food and water for 36 days. As evident in Fig 6A and 6B, by the end of the study the WT cells and Cas9 expressing MDA-MB-231 cells had grown significantly more than the 4a cells and the tumors had a mean weight of 537 ± 78 mg (*n* = 12) and 753 ± 168 mg (*n* = 10), respectively, compared to 197 ± 18 mg (*n* = 10) for the H_V_1 KO cells (*p*<0.01). The physical appearance of the tumors after excision was also notably different. Tumors isolated from H_V_1 KO cells were small and white with a solid center. In contrast, the tumors from Cas9 and WT cells were larger and often necrotic and pus-filled in the center. In 3 animals from the Cas9 and WT groups, the tumors were not localized but appeared diffuse across the abdomen. The luminescence from these animals was also diffuse and demonstrated signal distributed across the abdomen; in contrast, these features were not seen in any animal from the KO group. Although there were differences in the luminescence patterns of the WT and Cas9 groups compared to the H_V_1 KO group, the total integral of luminescence of the WT, Cas9, and 4a groups did not significantly differ (Fig 6C). Prepared and immunostained tissue from two tumors (WT vs. group 4a) selected as most representative of each group relative to the growth data obtained was processed for the histological identification of nuclear protein Ki67 (pKi67) reactivity (brown intracellular staining) (Fig 6D). The expression level of Ki67 is well characterized at the molecular level and is extensively used clinically as a prognostic and predictive marker for cancer diagnosis and treatment. Ki67 expression is strongly associated with tumor cell proliferation and growth, and is used in routine tumor evaluation as a cellular proliferation marker. Analysis of x 100 images for Ki67 positive cells demonstrated a reduction in the number of stained cells in the KO cells compared to both WT and CAS9 (Fig 6D). KO cells had 12.4 ± 1.1 stained cells per ROI (mean ± SEM, n = 12) compared to 25.6 ± 2.2 and 30.9 ± 3.7 for WT and Cas9 cells respectively (mean ± SEM, n = 12). Interestingly, the background hematoxylin and eosin staining also suggests qualitative differences in the nature of the cellular composition of the tumors, i.e., cellular density, morphology, and staining characteristics although this was not quantitatively pursued.

## Discussion

Here we examined several possible mechanisms of action of H_V_1 in breast cancer cells using the MDA-MB-231 cell line as a model system. The inspiration for the study was provided by studies by Wang et al. [21] reporting that proton channel expression in breast cancer cells was correlated with an increase in migration and an inferior clinical outcome. Expression data in the METABRIC study [48] suggest that increased levels of H_V_1 expression occur in approximately 3% of breast cancer patients (58 out of 1905 patients) and are clustered almost entirely in the molecular subset of Claudin-low (S1 Fig). Creation of Kaplan-Meier survival plots from data of patients in the METABRIC study who overexpressed H_V_1 did not show a significantly different survival rate compared to the general population of breast cancer sufferers, apparently in contradiction to the clinical data from the studies of the Wang et al. [22]. However, the interpretation of both sets of data is not straightforward for the following reasons. The Wang et al. studies were based entirely on immunohistological data using an antibody that they had generated that evidently has not been fully validated by them or others. We have evaluated 5 different H_V_1 antibodies obtained from commercial sources with western blotting, and none of them convincingly detected human H_V_1 protein. In the present study we used an antibody generously provided by a colleague (Dr. Melania Capasso, DZNE, Bonn, Germany), that we validated in-house by overexpression of H_V_1 in HEK cells (Fig 2C) using a plasmid that we have found to generate proton currents reliably [49]. It is conceivable that the Wang et al. antibody results reflect non-specific immunoreactivity unrelated to the expression of the proton channel. Conversely, the METABRIC study examines only mRNA at the cellular level; it is well-established that mRNA and protein levels are can be poorly correlated [50, 51]. Therefore, even though there is good evidence that HVCN1 mRNA was upregulated in the 58 patients from that study, no actual protein expression data exist to corroborate the mRNA expression.

Our data indicate that three breast cancer cell lines express H_V_1 that is readily detectable via western blotting, namely sum229PE, Hs578t, and MDA-MB-231. However, due to the low intensity of antibody staining and the absence of convincing patch clamp evidence of proton current we cannot confirm that the first two of these cell lines express sufficient levels of H_V_1 to affect cell function significantly. We chose to focus on MDA-MB-231 cells because H_V_1 expression was highest in this cell line and proton current was confirmed electrophysiologically. Nevertheless, a fraction of MDA-MB-231 cells lacked measurable H_V_1 current. Whether some cells in this cell line do not express H_V_1 at all or whether the expression level is low enough in some cells to prevent detection of current is unclear. In any case, the expression of H_V_1 in this cell line is heterogeneous, which might suggest the existence of different phenotypes within this cell line, a phenomenon previously noted [52]. Thus, the possibility that only a subset of these cells express H_V_1 cannot be excluded. Certainly, most of the MDA-MB-231 cells recorded here expressed *bona-fide* H_V_1. Unequivocal proton currents were measurable by the patch clamp technique and their properties, including opening kinetics, pH sensitivity, selectivity, and inhibition by Zn^2+^, all resemble analogous properties of H_V_1 in other mammalian cells (alveolar epithelial cells, neutrophils, eosinophils, macrophages, monocytes, sperm, oocytes, etc).

### An examination of the reactive oxygen species hypothesis

Our initial experiments repeated and extended data from the Wang et al. studies [21, 22]. In order to evaluate changes in motility of the cells, we used shRNA inhibitory sequences derived from the mission RNA database. Of 5 shRNA sequences evaluated, 2 were generally effective in reducing H_V_1 expression. Our wound scratch test experiment revealed that knockdown of H_V_1 inhibited the movement of cells over 24 hours (Fig 3A & 3B), consistent with the previous study. We postulated that changes in NADPH oxidase activity could be a plausible mechanism for the action of H_V_1 in these cells, because of close relationship between NADPH oxidase and H_V_1 in neutrophils, eosinophils, B-cells and monocytes where its role is well studied and established [11, 14, 53, 54]. We detected mRNA of NADPH oxidase variants, including Nox1, Nox2, Nox3, Nox5, Duox1, and Duox2 in these cells, which confirmed that the molecular machinery necessary for producing reactive oxygen species was present. Measuring the release of H_2_O_2_ from knockdown cells compared to a scrambled shRNA plasmid showed a significant reduction in the release of H_2_O_2_ although the release levels appeared to correlate with the level of H_V_1 expression only qualitatively. The cells transfected with the 2.1 plasmid did not show a significantly greater reduction in the release of H_2_O_2_ compared to those transfected with 3.1 plasmid even though the differences in H_V_1 protein expression are 5% and 24% of wild type respectively (Fig 3E).

### A comparison of knockdown (shRNA) and knockout (CRISPR/Cas9) of HVCN1

One might expect that functions inhibited by the knockdown of H_V_1 might show a greater level of inhibition if the channel were removed completely. Therefore, we created knockout cells using CRISPR/Cas9 to delete the HVCN1 gene. We targeted a sequence at the beginning of exon 2 of the gene to ensure that we removed expression of both long form and short form variants [20]. To achieve this, we first transfected Cas9 stably into MDA-MB-231 cells using Blasticidin as a selection tool and clonally isolated cells from the selection. Using these cells, we targeted the gene using a guide sequence that created several clones that lacked the expression and function of the proton channel as assessed by western blotting and patch clamp, respectively. We chose to use stably transfected cells using lentivirus because of the transfection efficiency and also because we had used lentiviral plasmids to transfect shRNA constructs for the KD experiments. It was felt that keeping molecular biological techniques consistent across cellular models would reduce any extraneous effects due to the particular treatment. However, we do not know where the different genetic sequences were inserted in the MDA-MB-231 genome.

Sequencing showed that Cas9 created a cut in the site specified and we used Cas9 cells as an additional control to wild type. Of the knockout cells isolated, many died shortly after creation with the clones 5f_2_ and 4a being the most stable; however only the 4a KO cells survived long enough to withstand labeling with luciferase and incorporation into a mouse xenograft model. We do not know why many of the clones died but it could be due to the transfection strategy employed utilizing successive lentiviral infections. Lentiviral transfection incorporates into the DNA based on a number of factors dependent on the cell’s current state of transcription [55]. Clones that died off may have had construct insertions creating a lethal phenotype. However, the clones we did use showed no obvious differences during tissue culture and seemed identical in cell morphology and growth rate. When we measured migration and H_2_O_2_ release from the knockout cells compared to the Cas9 control we did not observe inhibition of either migration or H_2_O_2_ release, in contrast to the results seen with shRNA knock-down of H_V_1. We chose to use Cas9 as a control because the Cas9 and KO cells had the initial lentiviral treatment and thus any differences between them would more likely be due to the deletion of H_v_1 rather than off target effects of viral transduction. Compared to WT MDA-MB-231 cells, H_2_O_2_ release was slightly lower, but Cas9 transfected cells generally had much lower H_2_O_2_ release than WT (reduced by 82% to 2.08 ± 0.5 μmols H_2_O_2_/1x10^6^ cells/hour, *n* = 10). Migration of the Cas9 cells was faster than WT cells and this phenotype was maintained in the KO cells where migration was not significantly different among Cas9, 4a and 5f_2_ cells (Fig 5A). The effects of Cas9 alone were unexpectedly large, and whatever mechanism was responsible, such as insertion of genetic constructs into genetic regions that might have interfered with H_2_O_2_ production.

We employed a reverse phase protein array available through MD Anderson Cancer Center (University of Texas; Houston, Texas) to screen differences in protein expression and phosphorylation of ~300 targets in the KO cells compared to WT and Cas9 (S2 Fig). L1CAM, an adhesion molecule that is associated with poor prognosis in certain breast cancers [56, 57], was expressed at much lower levels in the KO cells. We also detected a higher level of AKT phosphorylation (Ser473) in the KO cells. There was however a functional correlation with this increase in phospho-AKT, given that AKT phosphorylation plays a prominent role in the initiation of glycolysis [24], which is also consistent with the higher ECAR in the KO cells compared with WT, Cas9, and shRNA cells; regardless of mitochondrial inhibition.

Much of the evidence we accumulated indicated that knockdown and knockout cells functioned in qualitatively different fashions. Given that Wang et al. [21, 22] found that inhibiting the expression of H_V_1 with shRNA slowed tumor growth, we wondered whether the complete KO of H_V_1 from MDA-MB-231 cells would do the same. We chose WT and Cas9 cells as controls because the Cas9 had functional differences compared to the WT (smaller H_2_O_2_ release, faster migration) and the Cas9 cells were the parental clones of the KO cells. The intent was to minimize the changes attributable to the genetic modification. Consistent with expectations based on previous studies, the KO cells developed into significantly smaller tumors over 35 days compared to both WT and Cas9 cells (Fig 6A and 6B). Labeling the cells with a luciferase enzyme allowed us to monitor changes in luminescence via a bioanalyzer for the duration of the experiment. Taking the integral of luminescence, we found no significant difference between luminescence of the control compared to KO cells (Fig 6C). The reasons for this are not clear but may reflect the manner in which luminescence is generated. The luciferase reaction requires oxygen and thus changes in the perfusion of the tumors by blood can alter the signal. The smaller tumors of the KO group may have been better perfused than the WT and Cas9 cells, the latter resulting in a large tumor but with lower luminescence. Another possibility is that the overall number of tumor cells remained the same, but the WT and Cas9 cells tended to migrate away from their initial location. The resulting damage and necrosis in the inner regions of the tumor may have resulted in a necrotic tumor center with an outer layer of tumor cells that were similar in number compared to the KO tumors. In four animals injected with WT or Cas9 tumors the luminescence spread across the abdomen (Fig 6A) and in one case migrated from the injected side to the opposite mammary gland. There was the appearance of greater mobility of the control group luminescence in contrast with the very predictable size and location of luminescence of the KO tumors. The faster migration of Cas9 and WT cells in the scratch test (Figs 3A vs. 5A) seems to contradict the *in vivo* results. However, it would not be surprising if the processes involved in tumor invasion of surrounding tissues are distinct from those involved in cells repopulating empty regions of a flat surface.

In attempting to understand how H_V_1 might alter tumor growth, the decreases in H_2_O_2_ production and migration seen in the shRNA cells may not be causally relevant, because the KO cells showed neither of these traits yet exhibited significantly smaller tumor growth. Similarly, the significance of the increase in ECAR is complicated by the fact that our shRNA cells did not show the same phenotype yet siRNA treated cells in the Wang et al. study also exhibited reduced tumor growth.

A locally “inverted” acidic cellular environment seen in acidic tumors has been suggested to influence the invasive potential of tumor cells via the “acid-mediated invasion hypothesis” [45]. Gatenby and Gawinski proposed this hypothesis in 1996, describing the process of acid-mediated invasion as increased acid production serving as an intermediate by causing H^+^ flow down concentration gradients into adjacent normal tissue [58]. The chronic exposure of surrounding healthy tissue to an acidic microenvironment produces toxicity and cell death by the collapse of the transmembrane H^+^ gradient inducing necrosis or apoptosis [59]. The increased extracellular acidification enhances extracellular matrix degradation through the release of cathepsin B and other proteolytic enzymes. In contrast, tumor cells, due to their enhanced pH regulatory mechanisms, are resistant to the acid-induced toxicity, allowing them to survive and proliferate in the low pH microenvironments [60]. This permits the tumor cells to invade the altered adjacent normal tissue despite the acid gradients. *In vivo* experiments and modeling experiments have shown evidence for the presence of peritumoral acid gradients as well as cellular toxicity and extracellular matrix degradation in the normal tissue exposed to the acidic microenvironment [61]. Further evidence supporting this theory includes observations that neutralization of the tumor-derived acid with systemic buffers (bicarbonate) was sufficient to inhibit spontaneous and experimental metastases [62].

The presence of H_V_1 in late stage breast cancer at first glance appears to be in line with the upregulation of other pH transporters seen in cancer tissue. However, the mechanism of action of H_V_1 runs contrary to the development of an inverse proton gradient that precedes the invasion as predicted by the model of the acid-invasion hypothesis. Under the conditions of an inverted pH gradient the proton channel would be closed and therefore would be unlikely to affect tumor growth. The channel would open and function only if the cell were exposed to an outward pH gradient during its growth cycle. The evidence suggesting that H_V_1 enhances tumor growth and metastasis in breast cancer suggests that the acid-invasion hypothesis is not a complete picture of the factors that contribute to cellular invasion and tumor growth. To reconcile how H_V_1 works (it opens when the proton electrochemical gradient is outward and closes when the gradient is inwards) with the evidence that it is involved in tumor progression, we propose that the cell uses H_V_1 as a sensor of its external pH environment. This mechanism could explain the changes in protein expression in the absence of H_V_1 (downregulation of adhesion molecules and upregulation of metabolism). RPPA analysis and RNAseq analysis (S2 Fig; S1 Table) of the KO cells compared to WT and Cas9 reveal a number of genes with altered expression; including downregulation of L1CAM, ECAM, β-catenin and upregulation of AKT2 activation and increased AKT-p473. In conditions that result in H_V_1 opening, the increase in cell adhesion molecules would facilitate moving through the microenvironment; whereas when H_V_1 closes the cell increases its metabolism to enhance survival in an externally acidic environment. This idea is consistent with the observation that tumors grow slower in the absence of H_V_1. This hypothesis is strongly supported by the lower expression of Ki67 in tumors from the KO clone compared to the WT which demonstrates that the WT cells are more proliferative than the KO cells (Fig 6).

The loss of L1CAM in the knockout cells suggests another possible role of H_v_1 in these cells if we assume only a subset of cells in the population of MDA-MB-231 cells contain the proton channel. L1CAM is known to be associated with CD133 in ovarian cancer in a stem cell phenotype [63]. Cancer stem cells are small subsets of cells within tumor populations that influence tumorgenicity, have self-renewal and differentiation potential [64]. L1CAM has been implicated in the stem cells of other tumor types such as glioblastoma [65] and as a marker for pluripotent stem cells [66]. With the recent discovery of H_v_1 in human oocytes [67], it is tempting to speculate the role of H_v_1 as a stem cell marker or a regulator of stem cell function. It might function as a sensor for the microenvironment and modulate the differentiation of cells into invasive species based on the proton gradient. Natural variation in stem cell numbers could account for the variation seen in the WT and Cas9 tumor. However, we have little more than circumstantial evidence to support this concept and many more experiments would be required to prove this hypothesis. [65]

In summary, we show that H_V_1 contributes to the growth of tumors created by the injection of the triple negative breast cancer cell line MDA-MB-231 into the mammary fat pad of mice. We find no support for the hypothesis that ROS production mediates the role of H_V_1 in breast cancer. We propose that H_V_1 acts as a sensor for extracellular acidity and thereby modulates both metabolism and the expression of adhesion molecules.

## Supporting information

S1 Fig Data from Human studies shows the expression of HVCN1 is highest in triple negative breast cancer and enriched in Claudin-low subtype. Data compiled from RNAseq data from patients with an increased expression of HVCN1 as determined by a Z score of 2. Out of 1904 patients 58 had increased expression of HVCN1 mRNA. Most of these patients had the claudin low molecular subtype and it was seen mostly in grade 3 tumors. When looking at the receptor status 24 out of 58 were from triple negative breast cancers, 21 from ER containing breast cancers with or without PR or HER2 receptor/protein. The right graph shows the difference in overall survival compared to months survival for cells with high HVCN1 expression (red) and normal HVCN1 expression.
(TIFF)

S2 Fig Hv1 KO clones have similar changes in protein expression. RPPA analysis of WT MDA-MB-231 cells, MDA-MB-231 Cas9 containing cells, and HVCN1 KO 4a, 5f2 and 1fb. Top is a heat map of the 300+ proteins analyzed by the procedure. Below is a collection of protein targets that were found to be at least 10% different in the WT and Cas9 containing cells compared to 4a, 5f2 and 1fb.
(TIFF)

S1 Table RNAseq analysis of KO clones compared to WT and Cas9 shows patterns of gene expression changes. Excel file of RNAseq data of WT, Cas9, 5f2 and 4a cell types. The spreadsheet compares the expression of WT and Cas9 against the expression of genes in the 4a and 5f2. Genes that were increased greater than 2-fold in each sets of samples are listed. 1217 genes were reduced in expression and 745 were increased in expression using this analysis. These changes in expression included a downregulation of L1Cam.
(XLSX)

S1 Raw images (PDF)
