# Correction: Expression and function of voltage gated proton channels (H_v_1) in MDA-MB-231 cells

**DOI:** 10.1371/journal.pone.0235158

**Published:** 2020-06-17

**Authors:** The PLOS ONE staff

## Notice of republication

An incorrect version of the author byline was published in error. This article was republished on May 8, 2020 to correct for this error. Please download this article again to view the correct version.
