# Supplementary figures and images for: Expression and function of voltage gated proton channels (Hv1) in MDA-MB-231 cells
Source: PLoS One. 2020 May 6;15(5):e0227522. doi: 10.1371/journal.pone.0227522 (PMC7202653; doi:10.1371/journal.pone.0227522)

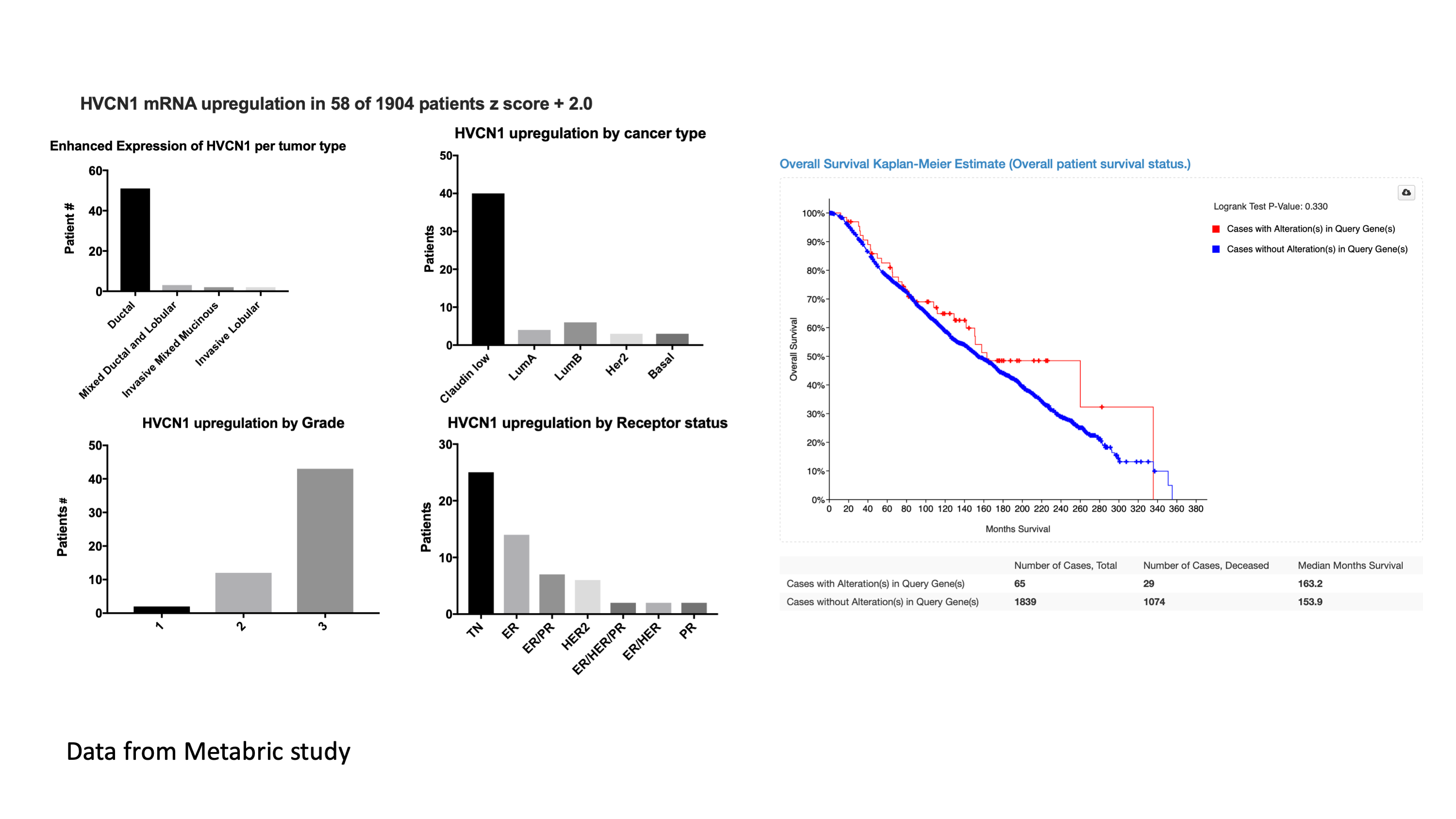

Supplement: S1 Fig — Data compiled from RNAseq data from patients with an increased expression of HVCN1 as determined by a Z score of 2. Out of 1904 patients 58 had increased expression of HVCN1 mRNA. Most of these patients had the claudin low molecular subtype and it was seen mostly in grade 3 tumors. When looking at the receptor status 24 out of 58 were from triple negative breast cancers, 21 from ER containing breast cancers with or without PR or HER2 receptor/protein. The right graph shows the difference in overall survival compared to months survival for cells with high HVCN1 expression (red) and normal HVCN1 expression. (TIFF) [file pone.0227522.s001.tiff]

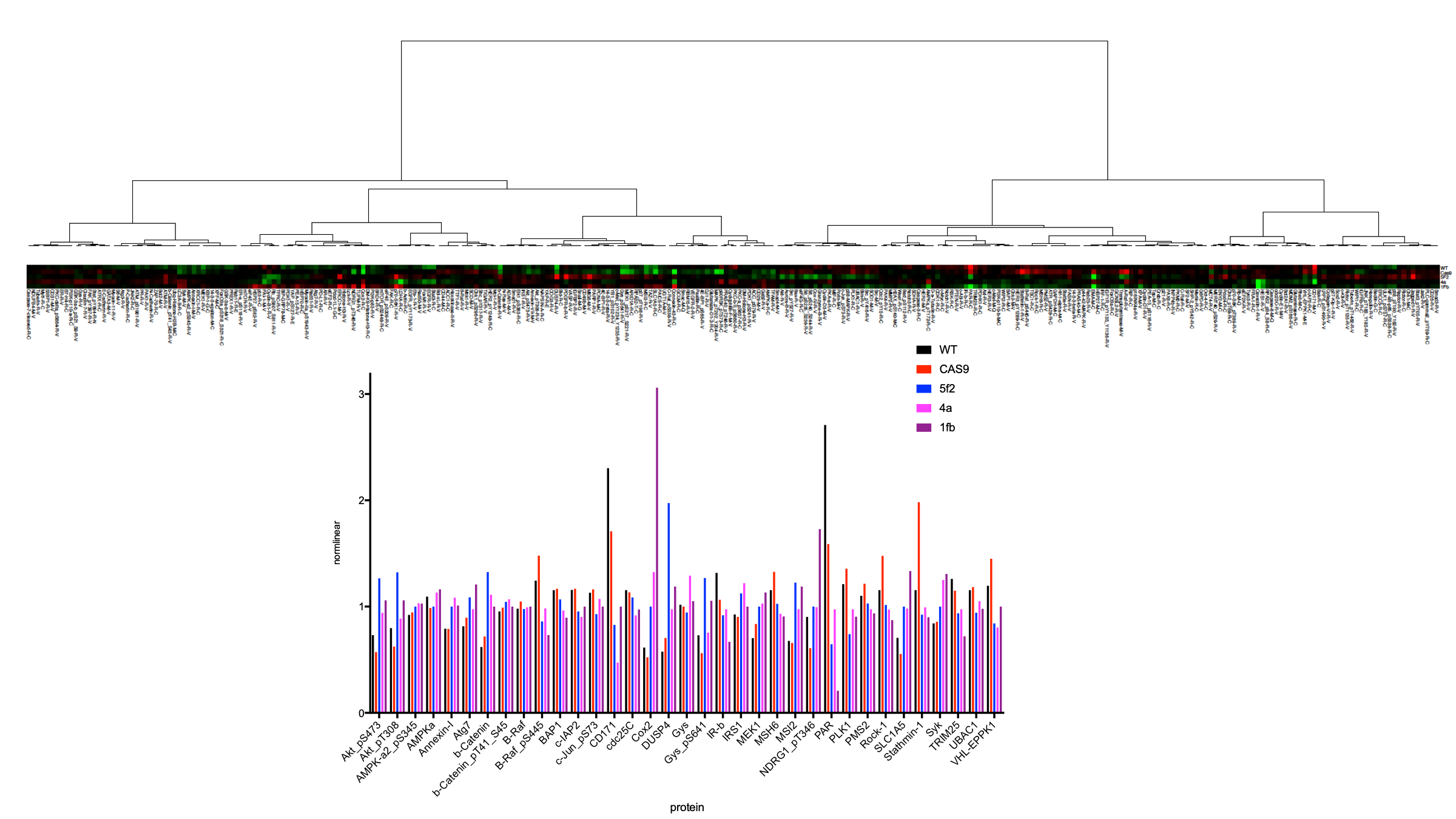

Supplement: S2 Fig — RPPA analysis of WT MDA-MB-231 cells, MDA-MB-231 Cas9 containing cells, and HVCN1 KO 4a, 5f2 and 1fb. Top is a heat map of the 300+ proteins analyzed by the procedure. Below is a collection of protein targets that were found to be at least 10% different in the WT and Cas9 containing cells compared to 4a, 5f2 and 1fb. (TIFF) [file pone.0227522.s002.tiff]
