# Supplementary material for: Expression and function of voltage gated proton channels (Hv1) in MDA-MB-231 cells
Source: PLoS One. 2020 May 6;15(5):e0227522. doi: 10.1371/journal.pone.0227522 (PMC7202653; doi:10.1371/journal.pone.0227522)
Supplement: S1 Raw images — (PDF) [file pone.0227522.s004.pdf]

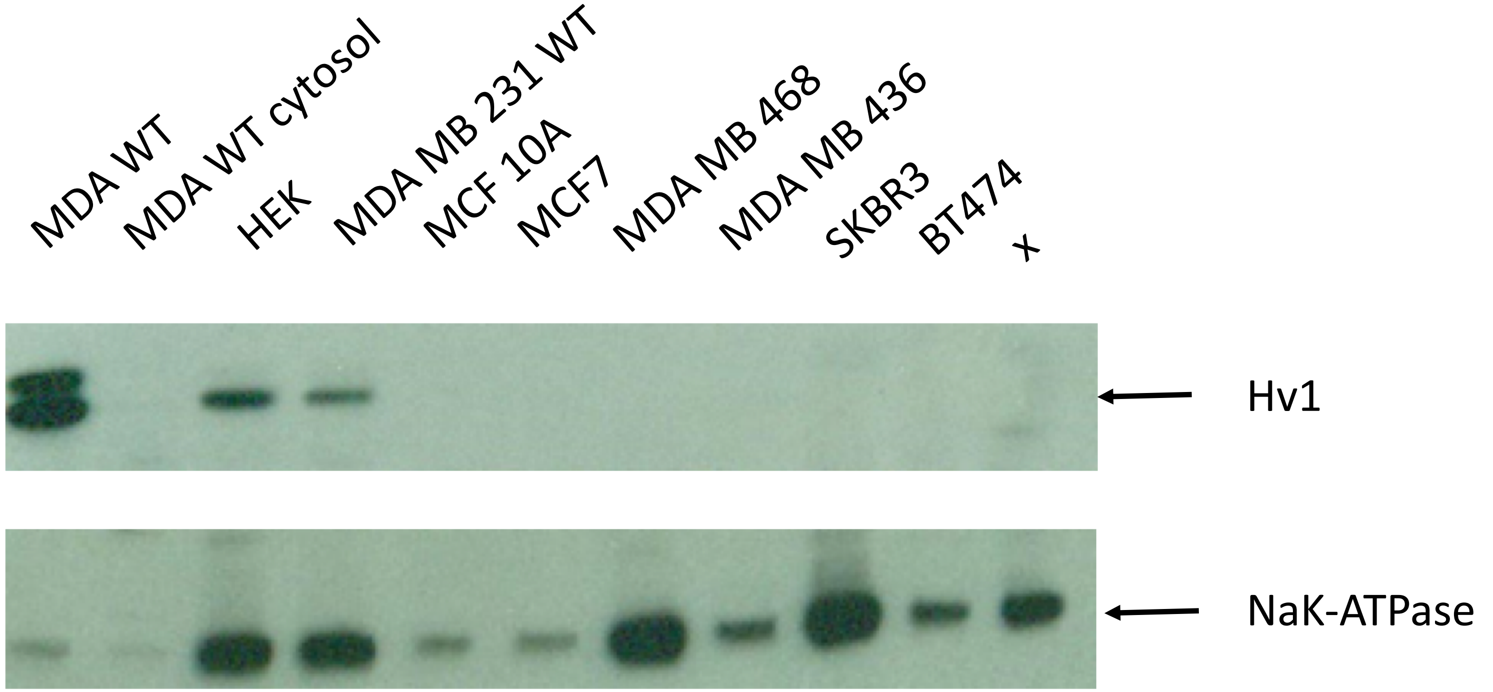
MDA WT
MDA WT cytosol
HEK
MDA MB 231 WT
MCF 10A
MCF7
MDA MB 468
MDA MB 436
SKBR3
BT474
x
Hv1
NaK-ATPase

Fig 2 c

Fig 2 d

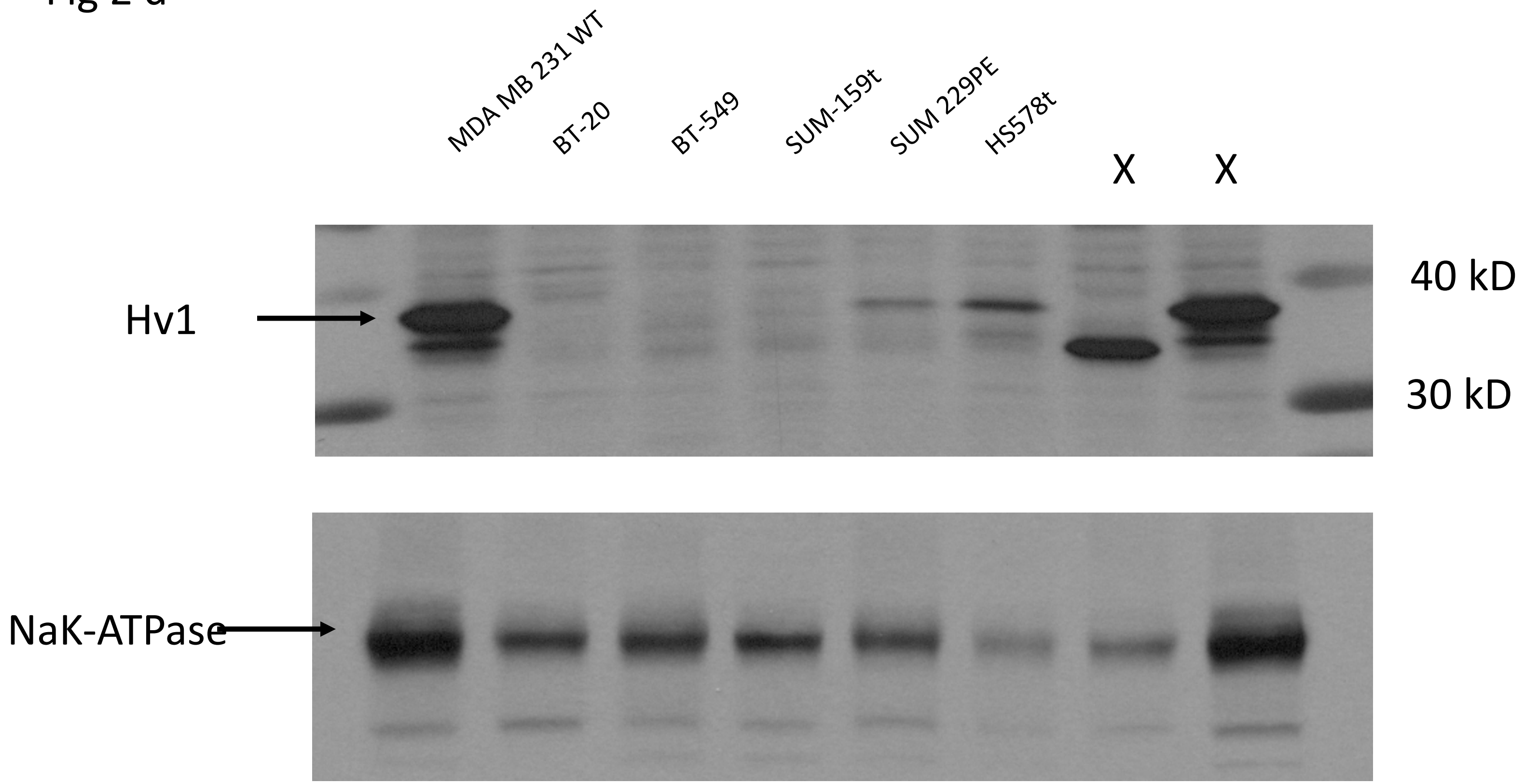
MDA MB 231 WT
BT-20
BT-549
SUM-159t
SUM 229PE
HS578t
X
X
Hv1
40 kD
30 kD
NaK-ATPase

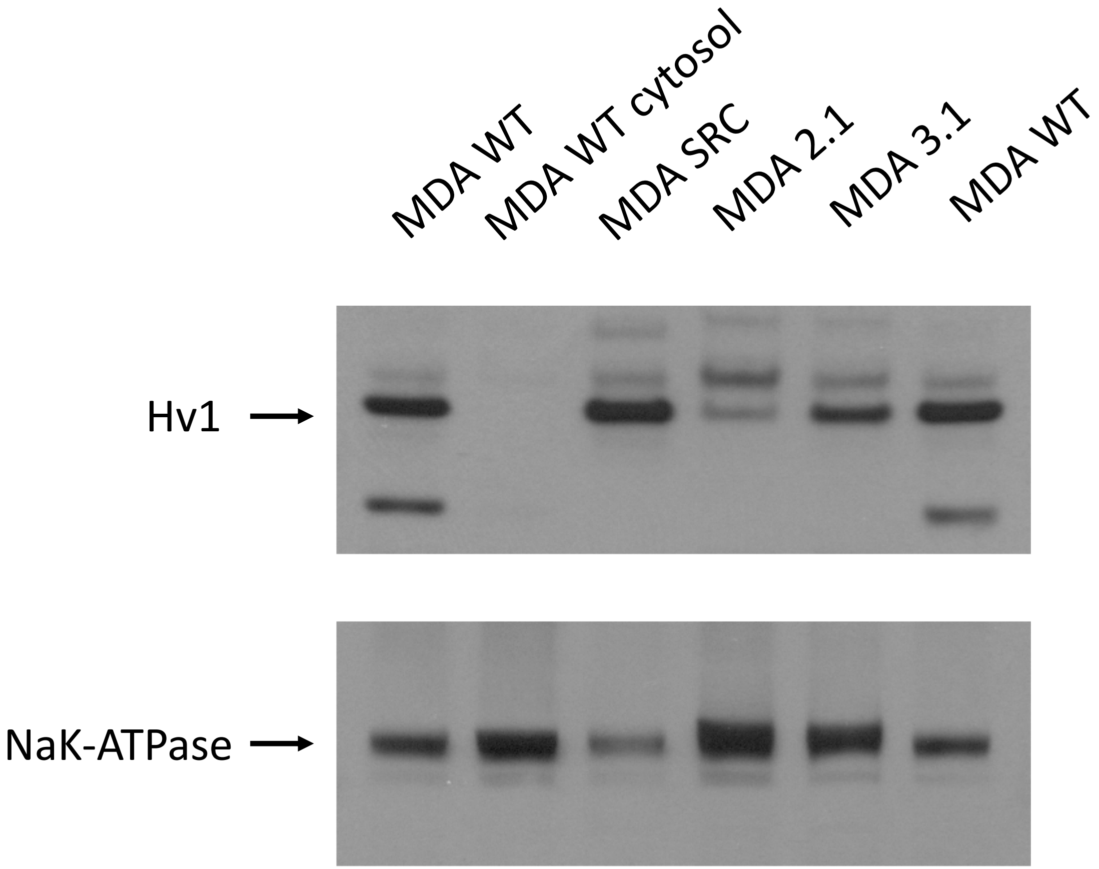
MDA WT
MDA WT cytosol
MDA SRC
MDA 2.1
MDA 3.1
MDA WT
Hv1
NaK-ATPase

Figure 3E

Figure 4a

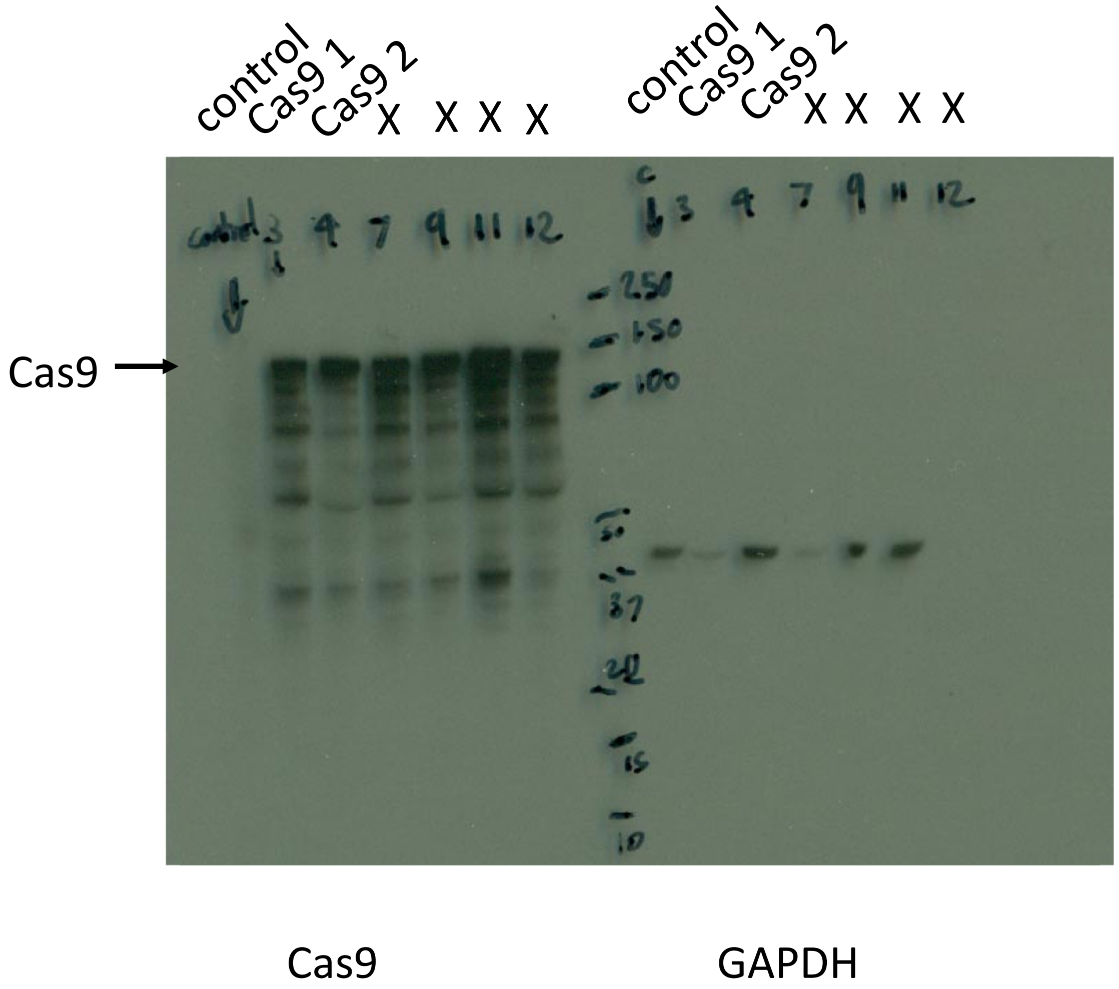

Figure 4b

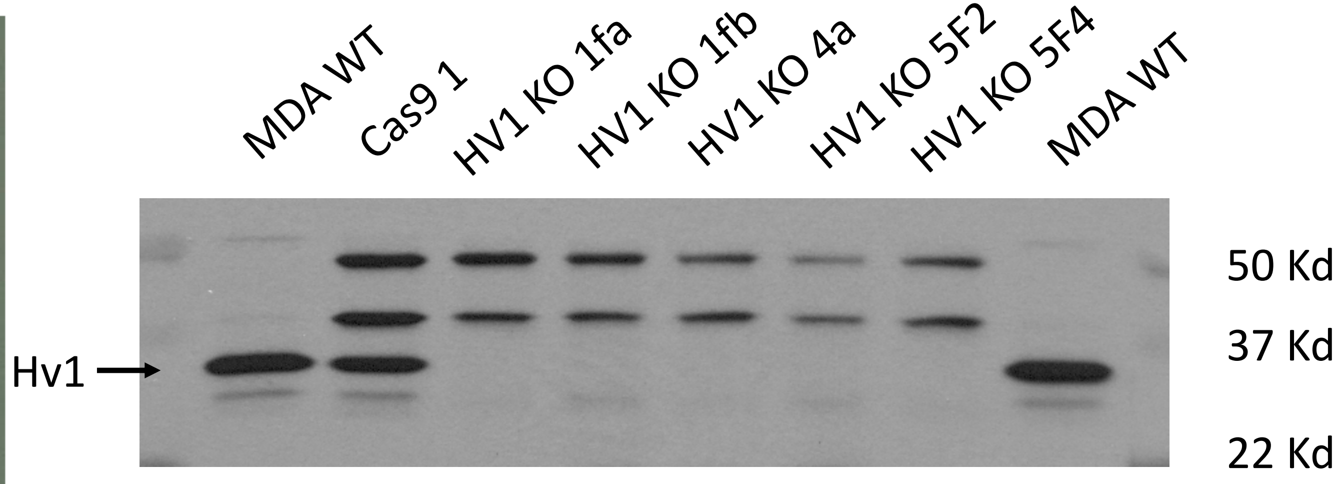

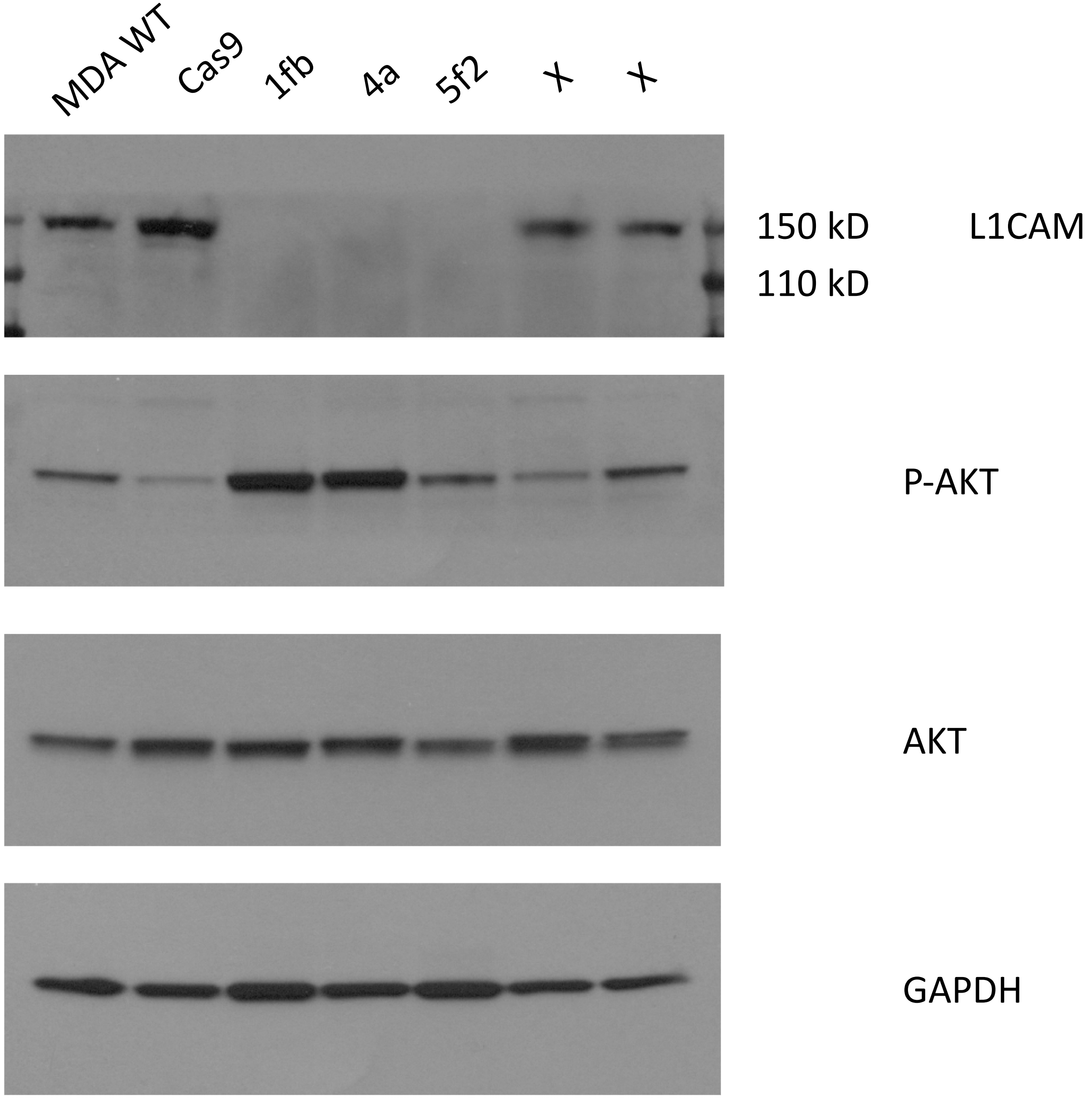

Figure 5F
